# Recombinant vesicular stomatitis virus–vectored vaccine induces long-lasting immunity against Nipah virus disease

**DOI:** 10.1172/JCI164946

**Published:** 2023-02-01

**Authors:** Courtney Woolsey, Viktoriya Borisevich, Alyssa C. Fears, Krystle N. Agans, Daniel J. Deer, Abhishek N. Prasad, Rachel O’Toole, Stephanie L. Foster, Natalie S. Dobias, Joan B. Geisbert, Karla A. Fenton, Robert W. Cross, Thomas W. Geisbert

**Affiliations:** 1 Galveston National Laboratory and; 2 Department of Microbiology and Immunology, University of Texas Medical Branch, Galveston, Texas, USA.

**Keywords:** Infectious disease, Vaccines, Adaptive immunity

## Abstract

The emergence of the novel henipavirus, Langya virus, received global attention after the virus sickened over three dozen people in China. There is heightened concern that henipaviruses, as respiratory pathogens, could spark another pandemic, most notably the deadly Nipah virus (NiV). NiV causes near-annual outbreaks in Bangladesh and India and induces a highly fatal respiratory disease and encephalitis in humans. No licensed countermeasures against this pathogen exist. An ideal NiV vaccine would confer both fast-acting and long-lived protection. Recently, we reported the generation of a recombinant vesicular stomatitis virus–based (rVSV-based) vaccine expressing the NiV glycoprotein (rVSV-*Δ*G-NiV_B_G) that protected 100% of nonhuman primates from NiV-associated lethality within a week. Here, to evaluate the durability of rVSV-*Δ*G-NiV_B_G, we vaccinated African green monkeys (AGMs) one year before challenge with an uniformly lethal dose of NiV. The rVSV-*Δ*G-NiV_B_G vaccine induced stable and robust humoral responses, whereas cellular responses were modest. All immunized AGMs (whether receiving a single dose or prime-boosted) survived with no detectable clinical signs or NiV replication. Transcriptomic analyses indicated that adaptive immune signatures correlated with vaccine-mediated protection. While vaccines for certain respiratory infections (e.g., COVID-19) have yet to provide durable protection, our results suggest that rVSV-*Δ*G-NiV_B_G elicits long-lasting immunity.

## Graphical abstract

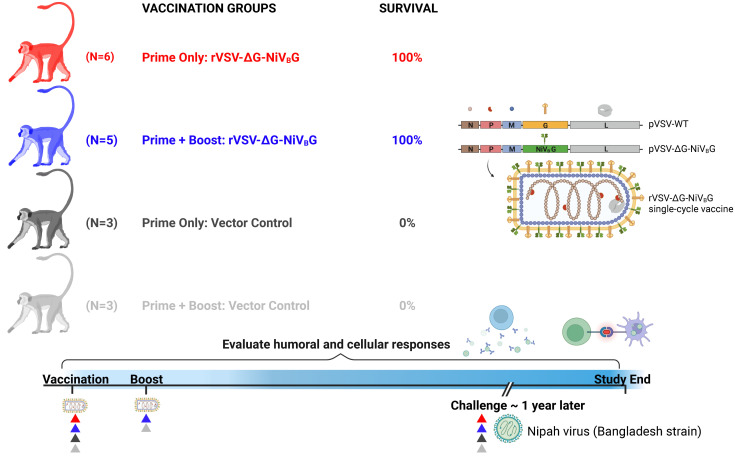

## Introduction

Nipah virus (NiV) causes encephalitis and severe respiratory disease in humans with case-fatality rates (CFRs) ranging from 40% to 100% (1). Inherent features of NiV engender concern that the virus could spark another pandemic. These attributes include (a) its ability to transmit via respiratory droplets, (b) its high mutation rate as an RNA virus, (c) its endemicity in regions with high population densities such as communities in South Asia, (d) the extensive geographic range of its natural reservoir (pteropid fruit bats), and (e) the near-annual outbreaks of NiV disease occurring in Bangladesh and India (2). For these reasons, NiV is included on the WHO’s Blueprint List of Priority Pathogens as well as the Coalition for Epidemic Preparedness Innovations (CEPI) Priority Pathogens List (3). In 2020, the US Centers for Disease Control and Prevention (CDC) proposed adding NiV to the Tier 1 Select Agents list because of its bioweapon potential (4); currently, NiV remains a non–Tier 1 CDC and US Department of Agriculture Select Agent (5).

Phylogenetic analyses indicate that at least 2 strains of NiV exist (6). The Malaysia strain (NiV_M_) caused the initial outbreak of NiV in Malaysia and Singapore resulting in over 270 cases and a CFR of 40%. The Bangladesh strain (NiV_B_) has caused repeated outbreaks in Bangladesh and India with an average CFR of approximately 75%. Although the genomes of these strains vary by less than 10% at the nucleotide level, NiV_B_ is more virulent than NiV_M_ under identical experimental conditions in nonhuman primates (NHPs) (7). A shorter therapeutic window also exists for NiV_B_, suggesting that medical countermeasures should be assessed against this more pathogenic strain (7).

Fast-acting and durable vaccines are desperately needed to combat NiV outbreaks. Several vaccines have shown promise in preclinical models, but no licensed vaccines or therapeutics are available for human use. Most vaccine candidates target the NiV surface glycoprotein (G) and/or fusion protein (F) as immunogens, as these proteins are required for virus entry and are readily recognized by the host immune system (8). For example, vaccinia and canarypox vectors encoding NiV_M_F or NiV_M_G have shown protection against NiV_M_ in hamsters and pigs (9, 10); a recombinant chimpanzee adenovirus vaccine expressing NiV_B_G completely protected hamsters against exposure to NiV_B_ and NiV_M_ (11); and a recombinant adenovirus-associated virus vaccine expressing NiV_M_G completely protected hamsters against a homologous NiV_M_ challenge (12). Other vaccine candidates include a virus-like particle–based NiV vaccine that protected hamsters against a homologous NiV_M_ challenge (13) and an mRNA vaccine encoding Hendra virus (HeV) G that protected 70% of hamsters and reduced viral load against a NiV_M_ challenge (14). The latter vaccine recently advanced to phase I clinical trials in humans (ClinicalTrials.gov NCT05398796), although its efficacy in the most stringent animal model, NHPs, has not yet been reported. As vaccine and therapeutic protection is generally more difficult to achieve in NHP versus rodent models, a demonstration of countermeasure efficacy in the NHP model is ideal. NHP testing is also useful for immunobridging purposes in the absence of human efficacy data.

African green monkeys (AGMs) are considered the “gold standard” NHP model for NiV as they most accurately mimic human disease (15). Some promising vaccines include a recombinant measles virus vector expressing NiV_M_ G that demonstrated partial efficacy in AGMs against NiV_M_ (16) and a Hendra virus G subunit vaccine (HeV sG) that fully defended AGMs against NiV_M_- and NiV_B_-associated lethality (17, 18). Alum-adjuvanted HeV sG is currently in phase I trials to assess its safety profile in humans (NCT04199169) (19). Another encouraging vaccine candidate effective against both NiV_M_ and NiV_B_ strains is based on the same recombinant vesicular stomatitis virus (rVSV) platform as the licensed vaccine rVSV-ZEBOV (Ervebo) (20–25). We recently reported the generation of a rapid-acting vaccine composed of a single-cycle rVSV expressing the Bangladesh strain glycoprotein of NiV (rVSV-ΔG-NiV_B_G) (26). VSV glycoprotein supplied in *trans* is necessary for replication of this vector; therefore, the vector is only capable of undergoing a single round of replication in susceptible cells. The highly attenuated nature of single-cycle rVSVs may be preferable to replication-competent rVSV vectors that are associated with vaccine-related adverse events in association with viral replication, including arthralgia/arthritis, dermatitis, and cutaneous vasculitis (27). Moreover, we have shown that a single-cycle vector was equivalent to a replication-competent VSV vector in generating high-level antibody responses to the NiV G (26, 28). In fact, in a previous study, serum neutralization titers were higher in AGMs vaccinated with rVSVs expressing G only versus F only or combined F and G (28). Similarly, higher anti–NiV G IgG and neutralizing antibodies were detected in ferrets vaccinated with an rVSV vaccine expressing only G as an immunogen compared with both F and G (25). Most vaccine candidates require multiple injections to confer protective efficacy, yet a single dose of rVSV-ΔG-NiV_B_G one week prior to NiV exposure protected 100% of AGMs and ferrets from lethality (25, 28). At 3 days after immunization, 66% of AGMs were protected, demonstrating the rapid immunostimulatory properties of the vaccine (26). Ervebo was found to decrease transmission to close contacts of infected individuals in a phase III ring vaccination trial in Guinea during the 2013–2016 West Africa and 2018–2020 Democratic Republic of the Congo Ebola virus outbreaks (29–31). Similarly, a reactive vaccination approach with rVSV-ΔG-NiV_B_G could help contain NiV outbreaks.

For outbreak management or a deliberate release, multiple injections of a vaccine over a prolonged period are neither practical nor economical. A single-dose vaccine providing long-lived protection is ideal to prevent future occurrences and flare-ups of NiV disease. To test the ability of rVSV-ΔG-NiV_B_G to elicit sustained immunity, we vaccinated AGMs one year before challenge with a uniformly lethal dose of NiV. Humoral and cellular responses were monitored over the course of the study. An assessment of durability is crucial for evaluating the suitability of any vaccine destined for clinical use.

## Results

### Study design and vaccination.

**Figure 1:**
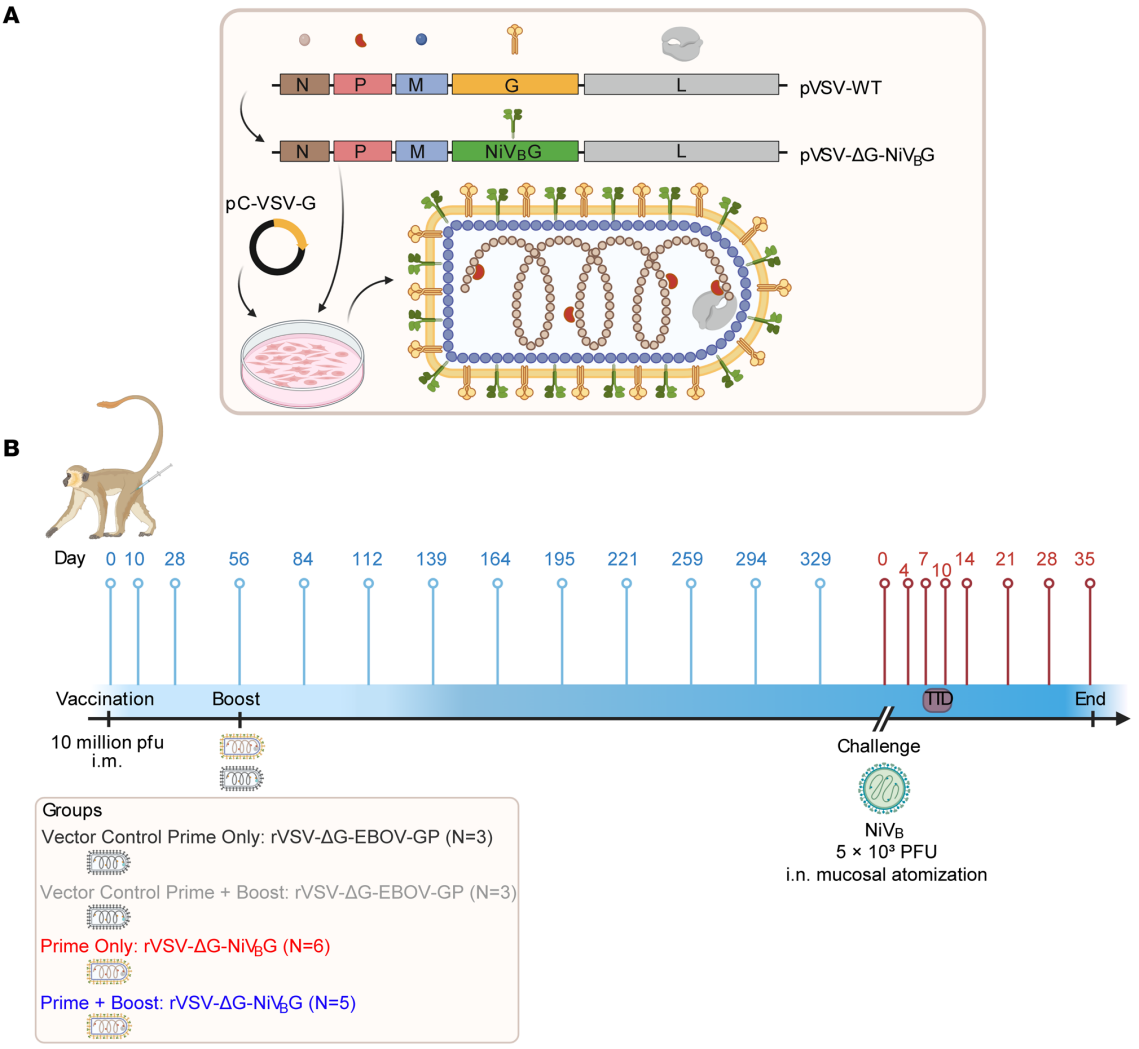
Vector and experimental design for the vaccination and challenge of AGMs. (**A**) Schematic of pVSV-WT and pVSV-ΔG-NiV_B_G genomes. The NiV_B_G gene (green box) was cloned into the native VSV G gene site (yellow box) in a plasmid containing the entire rVSV genome and recovered in VSV G–complemented (pC-VSV-G) baby hamster kidney cells. Intergenic and 3′- or 5′-untranslated genomic regions are indicated by black lines. (**B**) Seventeen AGMs were randomized into 4 groups: prime only (*n* = 6), prime + boost (*n* = 5), vector control prime (*n* = 3), and vector control prime + boost (*n* = 3) groups. Each group received a 1 × 10^7^ PFU i.m. dose of rVSV-ΔG-NiV_B_G vaccine or a nonspecific rVSV vector control expressing the Ebola virus glycoprotein (rVSV-ΔG-EBOV-GP). The prime + boost and vector control prime + boost groups received an additional dose at 56 days after vaccination. Blood samples were collected monthly at days 0, 10, 28, 56, 84, 112, 139, 164, 195, 221, 259, 294, 329, and 369 (0). AGMs were subsequently challenged 1 year later with an intranasal dose of 5 × 10^3^ PFU of NiV_B_ delivered by Mucosal Atomization Device. Post-exposure blood samples were collected at 4, 7, 10, 14, 21, 28, terminally, and/or 35 days. Blue pins indicate vaccination-phase sampling time points, whereas red lines denote challenge-phase sampling time points. N, nucleoprotein; P, phosphoprotein; M, matrix protein; G, glycoprotein; EBOV, Ebola virus.

We previously reported the generation of a single-cycle rVSV vaccine that elicited rapid protection against NiV disease (26) (Figure 1A). To evaluate the durability of rVSV-ΔG-NiV_B_G, we randomized 17 healthy adult AGMs into 4 groups: prime only (*n* = 6), prime + boost (*n* = 5), vector control prime (*n* = 3), and vector control prime + boost (*n* = 3) cohorts (Figure 1B). AGMs were intramuscularly vaccinated with a 1 × 10^7^ PFU dose of rVSV-ΔG-NiV_B_G or a nonspecific rVSV vector control expressing the Ebola virus glycoprotein (rVSV-ΔG-EBOV-GP). The prime + boost and vector control prime + boost groups received an additional dose of each respective vaccine at 56 days after vaccination (i.e., 28 days after prime immunization). To evaluate humoral and cellular response kinetics, blood samples were collected at least monthly on days 0, 10, 28, 56, 84, 112, 139, 164, 195, 221, 259, 294, 329, and prior to challenge. Approximately 1 year after administration of the first dose (369 days), all AGMs were challenged intranasally with a uniformly lethal dose of 5 × 10^3^ PFU of NiV_B_ as previously described (32). Post-exposure blood samples were collected at 4, 7, 10, 14, 21, 28, terminally, and/or 35 days.

### Survival and clinical signs.

**Figure 2:**
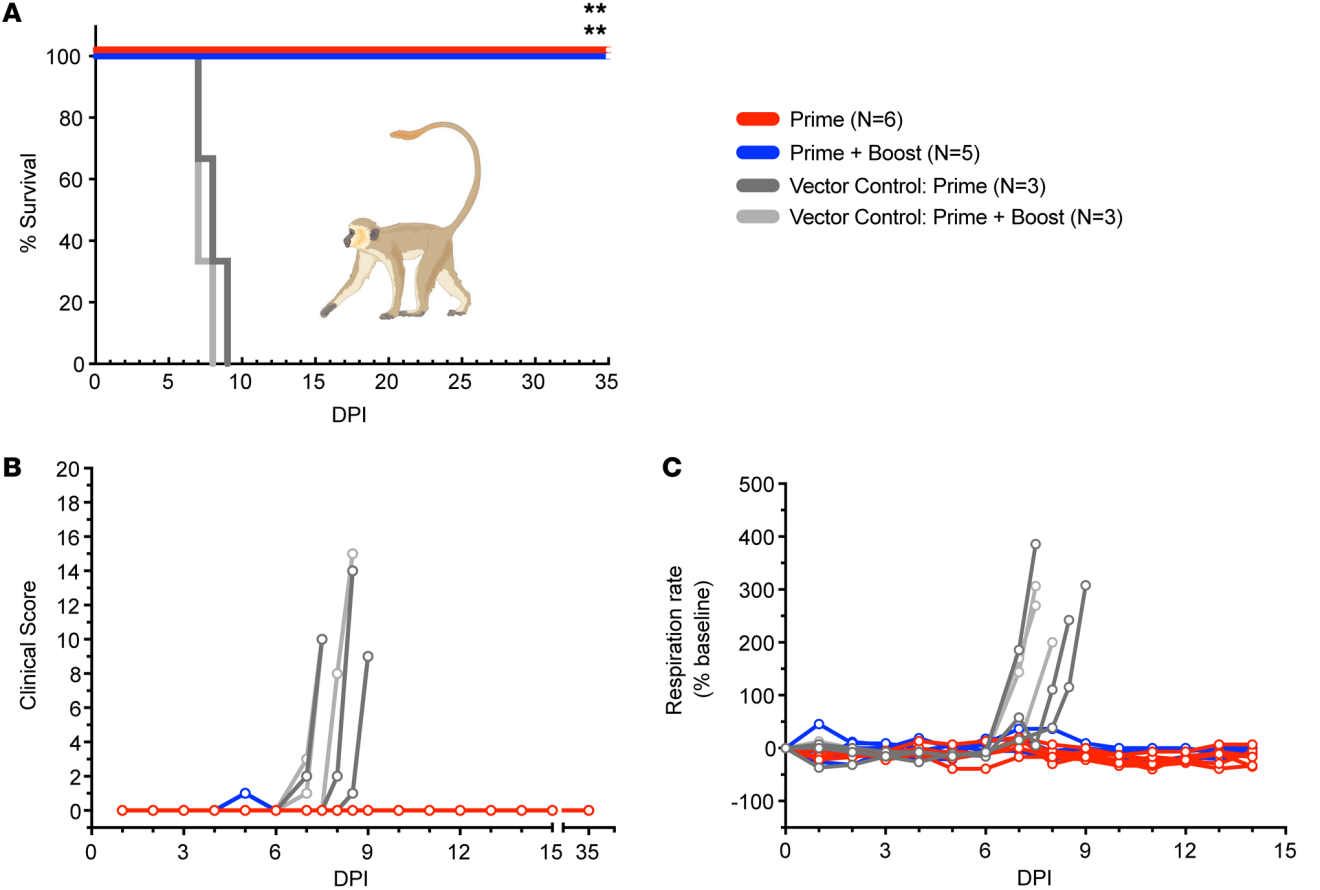
Survival and health of vaccinated AGMs exposed to NiV_B_. (**A**) Kaplan-Meier survival curves of vaccinated AGMs exposed to NiV_B_ for prime only (*n* = 6; red lines), prime + boost (*n* = 5; blue lines), vector control prime (*n* = 3; dark gray lines), and vector control prime + boost (*n* = 3; light gray lines) groups. A statistically significant association (log-rank test; **P < 0.0021) was found between prime and vector control prime, and prime + boost and vector control prime + boost, groups. (**B**) Clinical scores of individual AGMs vaccinated with rVSV-ΔG-NiV_B_G or a nonspecific rVSV vector control and challenged 1 year later with NiV_B_. (**C**) Respiration rates represent the percentage above or below baseline pre-vaccination values (beats per minute) of individual AGM subjects for each group challenged with NiV_B_.

Whether receiving a single dose of vaccine or prime-boosted, 100% of specifically immunized AGM subjects survived to the 35 days postinfection (DPI) study endpoint (Figure 2A). A statistically significant association was observed between prime and vector control prime groups (log-rank test; *P* = 0.0018) and prime + boost and vector control prime + boost groups (log-rank test; *P* = 0.0046), but not between prime and prime + boost groups. AGMs receiving a single dose or 2 doses of the nonspecific rVSV vaccine reached euthanasia criteria by 7–9 DPI. No clinical signs were evident in prime or prime + boost groups (Figure 2B) other than transient anorexia in subject PB-2 at 5 DPI (Table 1). Respiration rates did not significantly fluctuate from baseline levels in the groups vaccinated with rVSV-ΔG-NiV_B_G, whereas the vector control groups developed tachypnea and dyspnea indicating respiratory decline (Figure 2C).

Various hematological and serum biochemistry changes occurred throughout the vaccination and challenge phases of the study in all 4 cohorts (Table 1 and Supplemental Table 1; supplemental material available online with this article; https://doi.org/10.1172/JCI164946DS1). Clinical pathology in prime and prime + boost groups was mild, but some subjects exhibited decreased appetite as well as transient increases in alanine aminotransferase, aspartate transaminase, or γ-glutamyltransferase (Supplemental Table 1). After NiV_B_ exposure, all subjects in the vector control groups had elevated C-reactive protein values, indicating nonspecific systemic inflammation. All controls except VC-P-2 exhibited lymphocytopenia, and 4 of 6 subjects (VC-P-2, VC-P-3, VC-PB-1, VC-PB-2) developed thrombocytopenia (Table 1). Monocytosis, neutrophilia, and hypoamylasemia were also prominent findings in vector controls.

### Viral loads in vaccinated AGMs challenged with NiV_B_.

**Figure 3:**
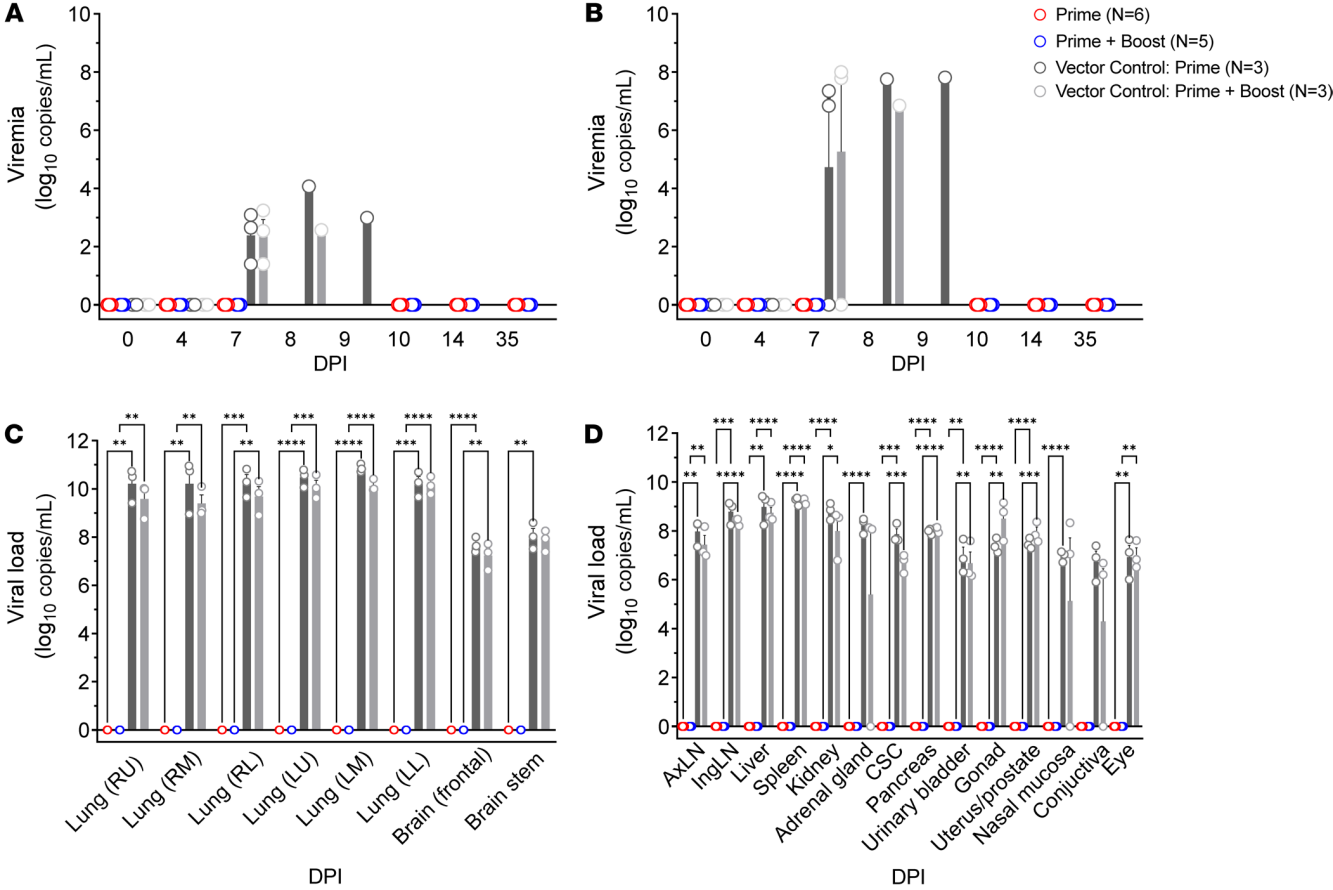
Viral loads of immunized AGMs after challenge with NiV_B_. Detection of NiV_B_ viral loads in EDTA plasma by plaque assay (**A**), whole blood by RT-qPCR (**B**), or tissues by RT-qPCR (**C** and **D**) for prime only (rVSV-ΔG-NiV_B_G; *n* = 6; red bars), prime + boost (rVSV-ΔG-NiV_B_G; *n* = 5; blue open circles), vector control prime (rVSV-ΔG-EBOV-GP; *n* = 3; dark gray bars), and vector control prime + boost (rVSV-ΔG-EBOV-GP; *n* = 3; light gray bars) groups. Bars represent the mean value for all members of the group at each time point, and upper error bars represent the SEM. Limit of detection (LOD) for plaque assays is 25 PFU; LOD for RT-qPCR is 1,000 copies/mL. Open circles represent average values from duplicates for individual subjects. Two-way ANOVA with Tukey’s multiple-comparison test; *P < 0.0332, **P < 0.0021, ***P < 0.0002, ****P < 0.0001. CSC, cervical spinal cord.

Viremia was undetectable by plaque assay or reverse transcriptase quantitative PCR (RT-qPCR) in subjects receiving a single dose or 2 doses of rVSV-ΔG-NiV_B_G (Figure 3, A and B). All vector controls, whether primed only or boosted, developed viremia following challenge. Shortly before euthanasia, infectious NiV_B_ titers in control subjects ranged from 2 to 4 log_10_ PFU/mL, while viral copies ranged from 6 to 8 log_10_ genome equivalents per mL.

Similarly, NiV_B_ replication in lung and neurological tissue (Figure 3C) or any other major organs (Figure 3D) was undetectable in all rVSV-ΔG-NiV_B_G–immunized subjects, indicating that specific vaccination induced sterilizing immunity. High viral loads were found in all organs tested for the vector control groups, including lymph node, liver, spleen, kidney, urinary, reproductive, and mucosal tissue (Figure 3, C and D).

### Pathology of vaccinated AGMs challenged with NiV_B_.

Necropsy was performed on AGMs after euthanasia. Lesions consistent with NiV disease were present in all vector controls (VC-P-1, VC-P-2, VC-P-3, VC-PB-1, VC-PB-2, VC-PB-3), including hemorrhagic pneumonia with extensive pleural effusion, hepatic congestion, splenomegaly, and meningeal congestion. Other lesions included adrenomegaly (VC-P-1, VC-P-2, VC-P-3, VC-PB-1, VC-PB-2, VC-PB-3) and lymphadenomegaly (VC-P-2). No gross lesions were apparent in specifically vaccinated subjects.

**Figure 4:**
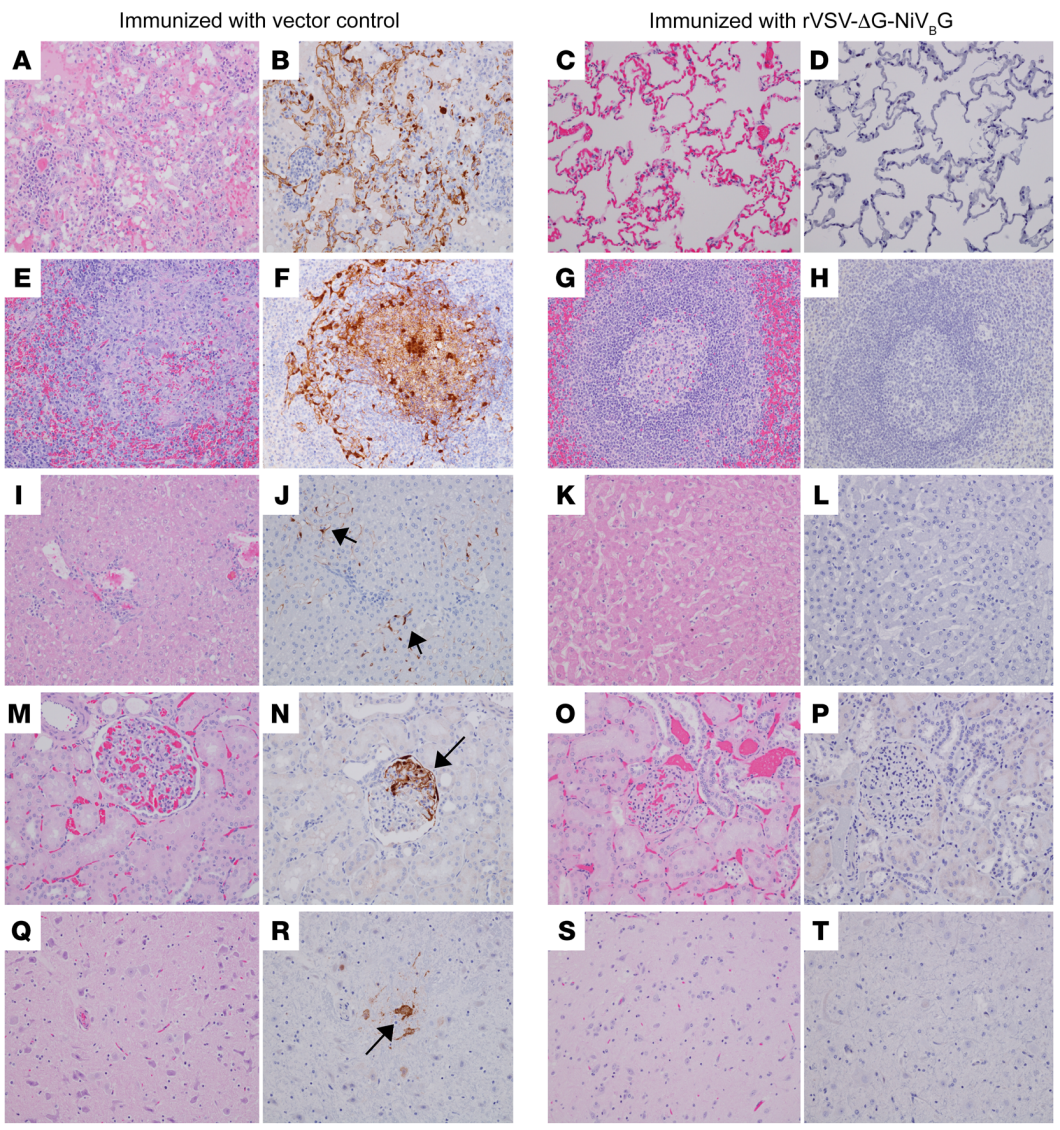
Pathology of vaccinated and control NiV_B_-infected AGMs. Representative photomicrographs of immunohistochemistry (IHC) for anti-NiV antigen (brown) in lung (**B** and **D**), spleen (**F** and **H**), liver (**J** and **L**), kidney (**N** and **P**), and brain (**R** and **T**); and H&E staining of the lung (**A** and **C**), spleen (**E** and **G**), liver (**I** and **K**), kidney (**M** and **O**), and brain (**Q** and **S**). All photomicrographs were taken at ×20 magnification. Micrographs shown are from positive controls VC-P-3 (**A**, **B**, **E**, **F**, **I**, **J**, **M**, and **N**) and VC-P-2 (**Q** and **R**). (**A**) Loss of normal pulmonary alveolar architecture with inflamed and necrotic alveolar septa and flooding of alveolar spaces with fibrin, edema, and hemorrhage. (**B**) IHC-positive endothelium and mononuclear cells within the alveolar septa and alveolar macrophages. (**E**) Loss of splenic germinal center architecture with lymphocytolysis, syncytial cell formation, and hemorrhage. (**F**) IHC-positive mononuclear cells concentrated in the white pulp and scattered within the red pulp. (**I**) Sinusoidal leukocytosis. (**J**) IHC positivity of sinusoidal lining cells and Kupffer cells (black arrows). (**M**) Renal glomerular congestion. (**N**) Segmental IHC-positive glomerular endothelium and mononuclear cells (black arrow). (**Q**) Diffuse gliosis of the brainstem. (**R**) IHC-positive neuronal cells of the brainstem (black arrow). No appreciable immunolabeling or lesions were noted in the lung, spleen, liver, kidney, or brain of representative rVSV-ΔG-NiV_B_G–surviving AGM P-3 (**C**, **D**, **G**, **H**, **K**, **L**, **O**, **P**, **S**, and **T**).

Prominent histological findings (Figure 4, A–R) in examined vector control sections included interstitial pneumonia (characterized by the expansion of alveolar septa with mixed mononuclear inflammation, edema, hemorrhage, and, in some areas, necrotic cellular debris) and flooded alveolar spaces (with numerous mononuclear cells, edema, hemorrhage, and occasionally necrotic debris merged imperceptibly with the remnants of the alveolar septa) (Figure 4A). Immunohistochemistry (IHC) positivity was prevalent in the endothelium lining alveolar septa, the endothelium of medium-caliber vessels, mononuclear cells within the alveolar septa, alveolar macrophages, and, rarely, the smooth muscle of large-caliber vessels (Figure 4B). Loss of typical germinal center architecture of the spleen was evident in vector control sections with a reduced lymphocyte population, flooding of white pulp with hemorrhage, and the presence of multinucleated cells (Figure 4E). Within the white pulp and scattered throughout the red pulp of the spleen, antigen reactivity was concentrated within mononuclear cells and syncytial cells (Figure 4F). Other common lesions in vector controls included lymphoid medullary histiocytosis (axillary and inguinal), sinusoidal leukocytosis of the liver (Figure 4I), nephritis (Figure 4M), adrenalitis, mild gliosis of the brain (Figure 4Q), esophagitis, tracheitis, myocarditis, cystitis, and, rarely, prostatitis. In these examined sections, anti-NiV immunolabeling was predominantly in mononuclear inflammatory cells and the endothelium. At the study end point, we were unable to detect substantial immunolabeling or ostensible histologic findings in corresponding tissues from rVSV-ΔG-NiV_B_G–primed or prime-boosted subjects (Figure 4, C, D, G, H, K, L, O, P, S, and T) or any other examined tissue: cervical spinal cord, pituitary gland, trigeminal ganglion, brain (frontal), brainstem, hippocampus, trachea/esophagus, heart, pancreas, urinary bladder, gonad, uterus/prostate, conjunctiva, nasal tissue, and eye.

### Neutralizing and anti–NiV_B_ G binding antibody titers in vaccinated AGMs.

**Figure 5:**
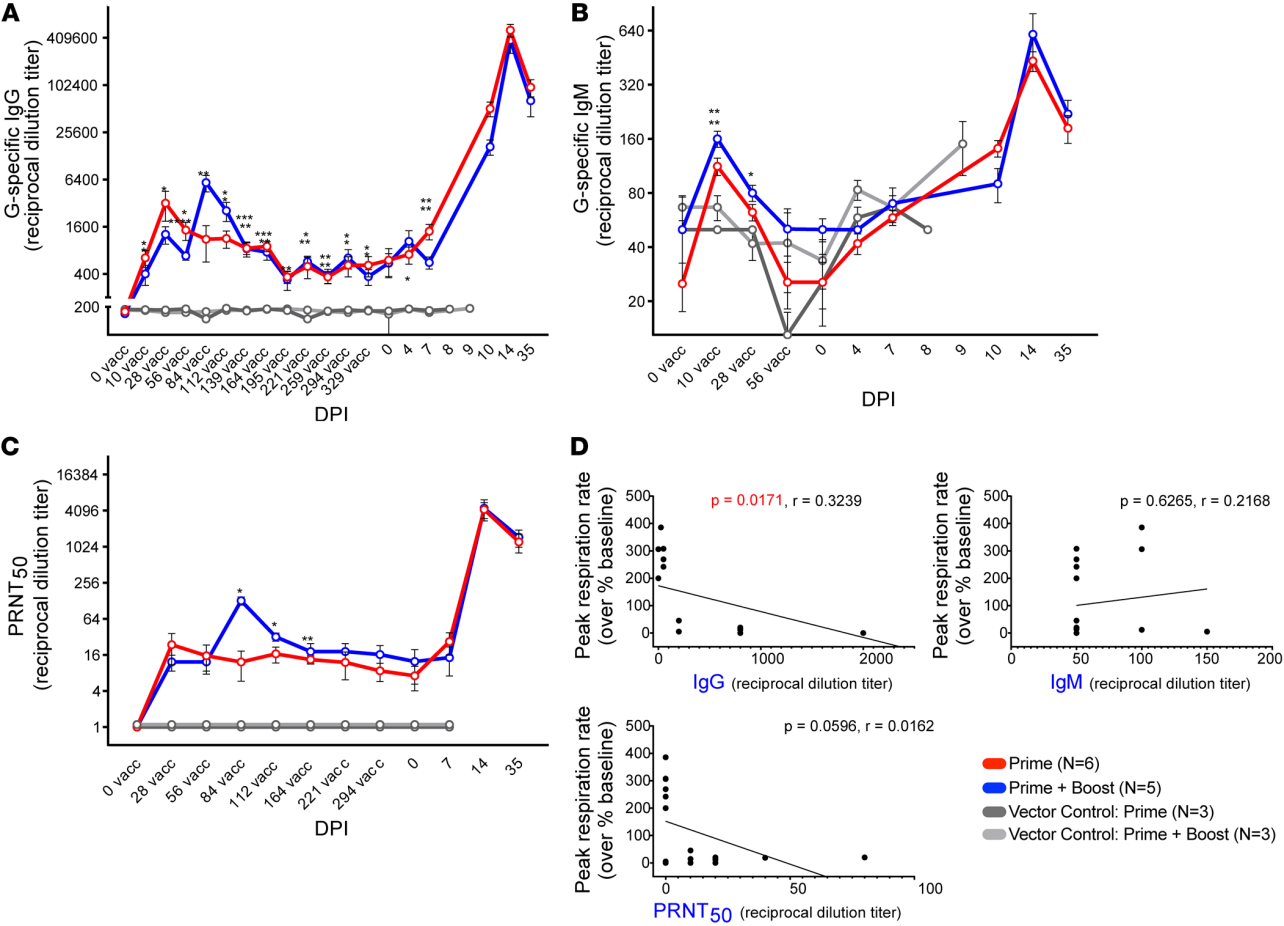
Humoral responses in vaccinated AGMs. (**A** and **B**) AGM serum samples were tested for circulating NiV G–specific IgG (**A**) and IgM (**B**) by indirect ELISA. Line graphs depicting the average reciprocal dilution titer for each group ± SEM (error bars) at each time point are shown. (**C**) The average anti-NiV neutralizing antibody titer for each group ± SEM (error bars) at each time point. PRNT_50_ values represent the reciprocal dilution at which plaque counts were reduced by 50% in comparison with control wells. Each group is denoted by line color: prime only (rVSV-ΔG-NiV_B_G; *n* = 6; red), prime + boost (rVSV-ΔG-NiV_B_G; *n* = 5; blue), vector control prime (rVSV-ΔG-EBOV-GP; *n* = 3; dark gray), and vector control prime + boost (rVSV-ΔG-EBOV-GP; *n* = 3; light gray). (**D**) Correlation plots for respiration rates versus IgG, IgM, and neutralizing antibody levels. G, NiV_B_ glycoprotein; PRNT, plaque reduction neutralization test; vacc, vaccination. Two-way ANOVA with Tukey’s multiple-comparison test; *P < 0.0332, **P < 0.0021, ***P < 0.0002, ****P < 0.0001. A Pearson test was used to determine correlations.

To measure the magnitude and persistence of the humoral response following rVSV-ΔG-NiV_B_G vaccination, we performed indirect ELISAs over the span of a year on serum from immunized AGMs. Neutralizing responses were evaluated by plaque reduction neutralization tests (PRNT_50_). Anti–NiV G IgG (1:50 to 1:1,600) (Figure 5A) and IgM (1:50 to 1:200) (Figure 5B) binding titers were generated by 10 days after vaccination. A high level of background was observed for IgM responses in control vector recipients. IgM responses waned by 56 days after immunization, while IgG responses slightly decreased but remained stable up until challenge. For IgG responses, the rVSV-ΔG-NiV_B_G–boosted group only appeared more immunogenic (repeated-measures ANOVA with Tukey’s test; *P* = 0.0345) at 84 days after vaccination (28 days past the booster dose) but by 5–6 months showed antibody levels resembling those in the prime-only cohort. All survivors (Figure 5C) developed neutralizing antibodies that persisted for at least 1 year. Robust anamnestic binding IgG (1:51,200 to 1:819,200) and neutralizing titers (1:640 to 1:10,240) were generated following NiV exposure. In contrast, neither the vector control primed nor boosted group developed substantial levels of binding or neutralizing antibodies. A negative association was found between respiration rate and IgG levels (Pearson correlation; *P* = 0.0171, *r* = 0.3239), but not IgM or neutralizing titers, indicating the importance of this isotype in mediating respiratory protection (Figure 5D).

### Cellular responses in vaccinated AGMs.

**Figure 6:**
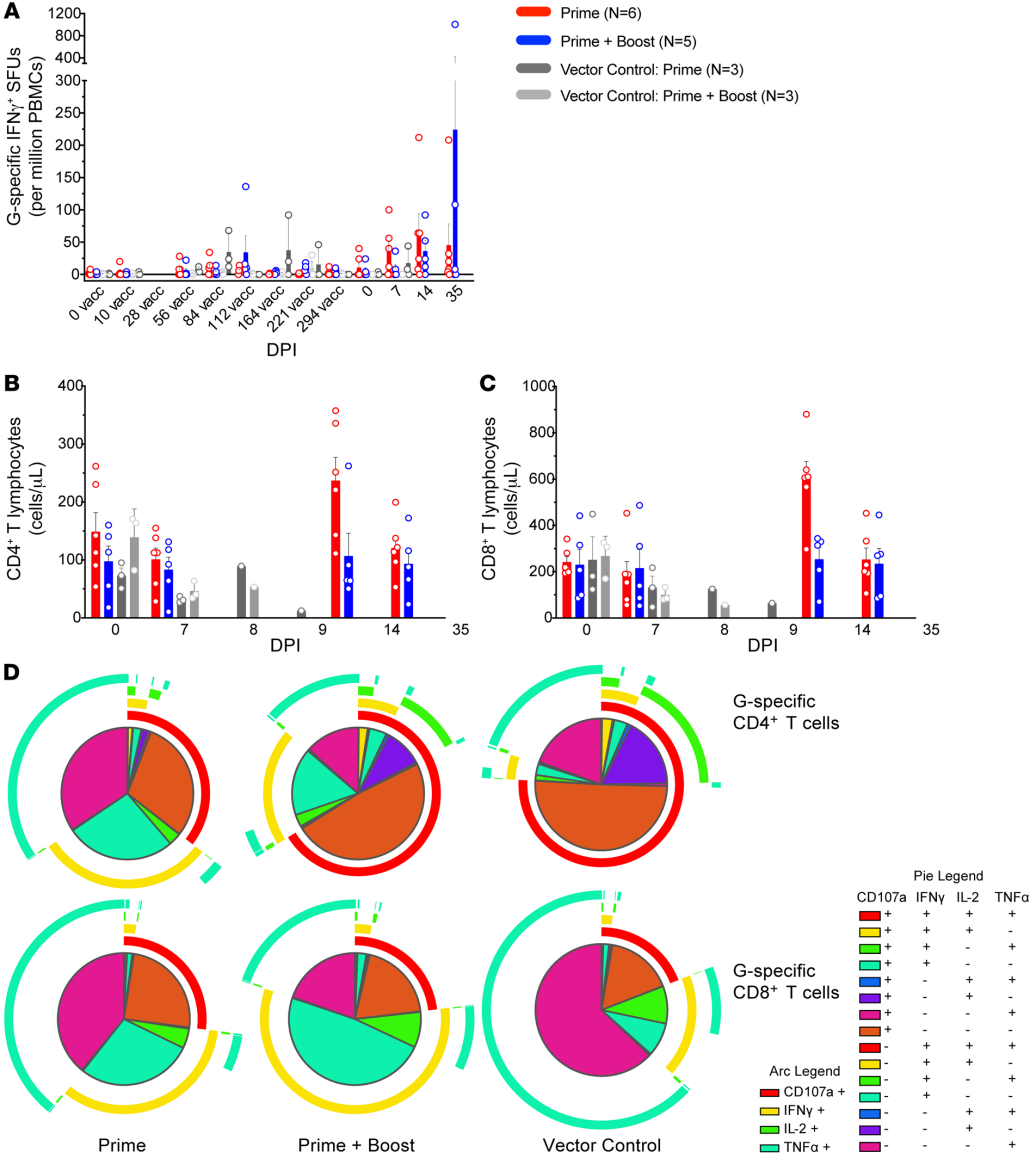
Cellular responses in immunized AGMs. (**A**) NiV G–specific IFN-γ^+^ spot-forming units (SFUs) in PBMCs from vaccinated AGMs for each group. Values were calculated by subtraction of the number of average spots from unstimulated duplicate wells from its respective stimulated counterpart at the corresponding DPI. A single replicate was excluded for 294 days after vaccination for subject P-B-1. (**B** and **C**) CD4^+^ (**B**) and CD8^+^ (**C**) T cell counts in vaccinated AGM PBMCs at each time point. Each group is denoted by the following: prime only (rVSV-ΔG-NiV_B_G; *n* = 6; red bars), prime + boost (rVSV-ΔG-NiV_B_G; *n* = 5; blue bars), vector control prime (rVSV-ΔG-EBOV-GP; *n* = 3; dark gray bars), and vector control prime + boost (rVSV-ΔG-EBOV-GP; *n* = 3; light gray bars). Bars represent the mean value for all members of the group at each time point, and error bars represent the SEM. Open circles represent the average value of duplicates from individual subjects. (**D**) Pie graphs depicting NiV G–specific CD4^+^ (top row) and CD8^+^ T cell (bottom row) cytokine profiles in PBMCs from each respective AGM group. The arcs denote the total percentage of degranulating (red), IFN-γ^+^ (yellow), IL-2^+^ (green), and TNF-α^+^ (teal) T cells. Each slice represents a specific combination of these markers. The vector control groups were combined for this analysis. G, NiV_B_ glycoprotein.

Cellular responses were evaluated over the course of the study by ELISPOT. Minimal NiV G–specific responses were evident in vaccinated AGM peripheral blood mononuclear cells (PBMCs), particularly during the vaccination phase. Starting at 7 DPI, low cellular responses were detected in approximately half of prime-only vaccinated subjects (P-1, P-3, P-5), 1 prime-boosted subject (PB-3), and 1 of the vector controls (VC-PB-3) (Figure 6A). By 14 DPI, G-specific IFN-γ spot-forming units (SFUs) were visible in wells containing PBMCs from all specifically vaccinated AGMs (range of 8 to 212 SFUs per million PBMCs) except for subjects P-6 and PB-3.

For a more granular analysis, we performed intracellular cytokine staining via flow cytometry to examine the polyfunctionality of CD4^+^ and CD8^+^ antigen-specific T cells in each AGM group. Our analyses indicated that specifically vaccinated subjects, whether receiving a single dose or 2 doses, had higher frequencies of CD3^+^CD4^+^ (Figure 6B) and CD3^+^CD8^+^ T cells following NiV exposure (Figure 6C). Moreover, prime-only and prime-boosted versus vector control subjects exhibited a higher degree of polyfunctionality and expressed more IFN-γ for both CD4^+^ and CD8^+^ G-specific T cell subsets (Figure 6D).

As natural killer (NK) cells are implicated in rVSV-mediated protection (26, 33–35), the functional capacity of this subset was also surveyed for each cohort. Specifically immunized subjects expressed higher frequencies of total (CD3^–^CD8a^+^) (Supplemental Figure 1A), degranulating (CD3^–^CD8a^+^CD107^+^) (Supplemental Figure 1B), and IFN-γ–secreting (CD3^–^CD8a^+^IFN-γ^+^) NK cells (Supplemental Figure 1C). Instead, vector controls exhibited an overall decline in these cell populations at late disease.

### Circulating cytokine detection.

As expected, the vector controls expressed higher levels of proinflammatory plasma cytokines and growth factors following NiV_B_ exposure, which is in line with ongoing viral replication and clinical disease in these subjects. At 7 DPI, significantly elevated inflammatory mediators in the vector control groups included inflammatory protein-10 (IP-10), monocyte chemoattractant protein-1 (MCP-1), and IFN-γ (Supplemental Figure 2, A–E). Conversely, higher levels of the monocyte chemoattractant IL-8 were observed in prime and prime + boost groups.

### Transcriptional correlates of protection.

**Figure 7:**
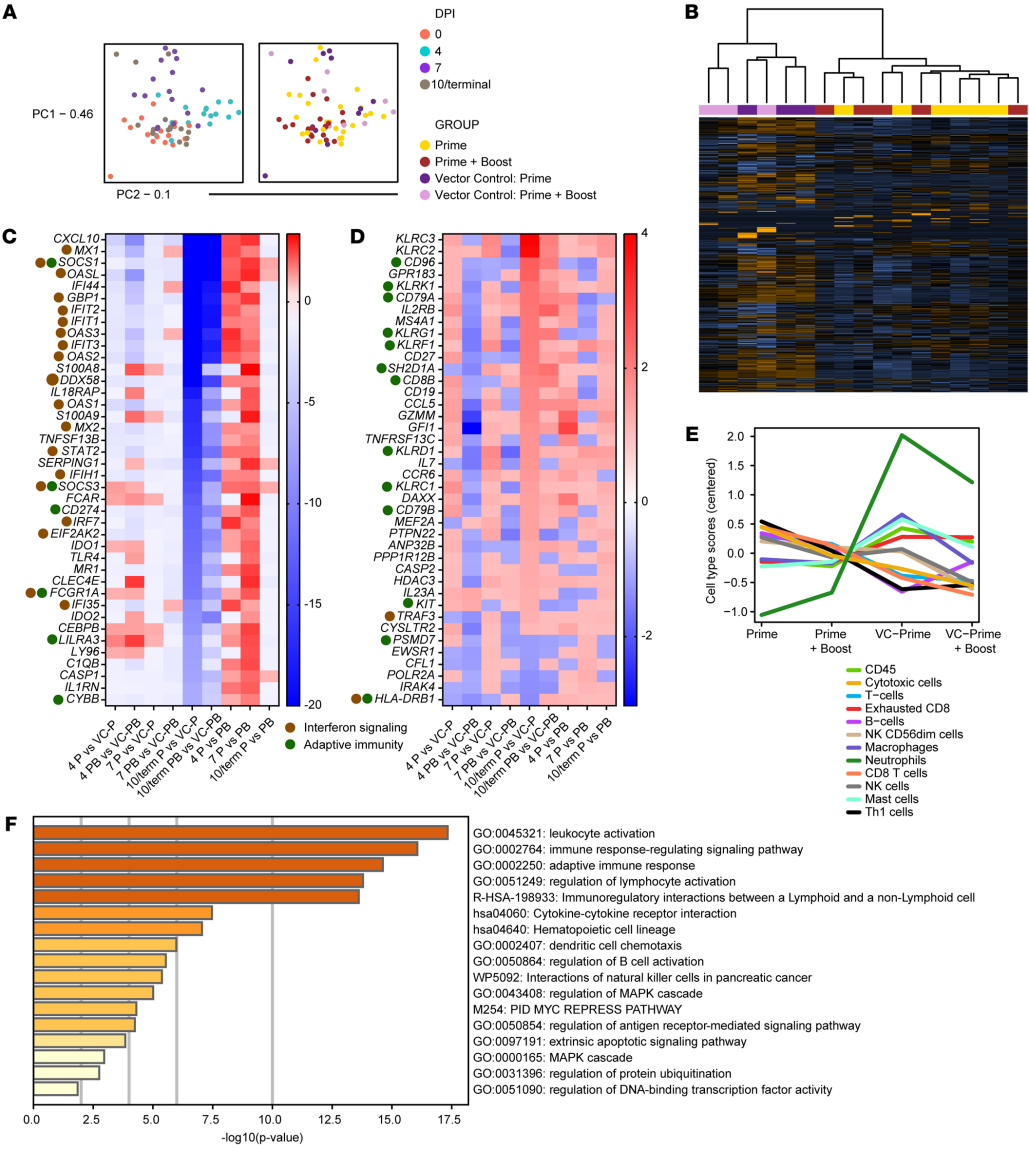
Transcriptional responses in AGMs after challenge with NiV_B_. (**A**) Principal component analysis based on DPI (0, 4, 7, 10/terminal time points) and each group: prime only (rVSV-ΔG-NiV_B_G; *n* = 6; yellow), prime + boost (rVSV-ΔG-NiV_B_G; *n* = 5; maroon), vector control prime (rVSV-ΔG-EBOV-GP; *n* = 3; purple), and vector control prime + boost (rVSV-ΔG-EBOV-GP; *n* = 3; lavender). (**B**) Overall expression changes for each group at late disease (orange denotes upregulated transcripts; blue denotes downregulated transcripts; black denotes no expression change). (**C** and **D**) Heatmaps depicting the topmost downregulated (**C**) and upregulated (**D**) transcripts in specifically versus nonspecifically prime-only vaccinated subjects at late disease (Benjamini-Hochberg–adjusted *P* value < 0.05). A comparison of prime versus boosted subjects was also performed. Dots indicate transcripts mapping to interferon signaling (brown) and adaptive immunity (green) nSolver gene sets. In the heatmaps, red denotes upregulated transcripts, blue denotes downregulated transcripts, and white denotes no expression change. (**E**) Trend plot depicting overall nSolver-derived cell-type quantities in control and vaccinated (fatal or survivor) cohorts. (**F**) Pathway enrichment of differentially expressed transcripts (Benjamini-Hochberg–adjusted *P* value < 0.05) in specifically vaccinated subjects at late disease. Displayed are the mean −log_10_(*P* values). A Benjamini-Hochberg test was used to derive adjusted *P* values. PC1, principal component 1; PC2, principal component 2.

To dissect molecular signals correlating with rVSV-ΔG-NiV_B_G–mediated protection, we performed targeted transcriptomics on peripheral whole blood RNA collected from AGMs. Dimensional reduction via principal component analysis revealed that variation in the data set was mostly driven by time point (0, 4, 7, 10 DPI or the terminal time point in fatal cases) rather than group (prime only, prime + boost, vector control prime, and vector control prime + boost) (Figure 7A). At 10 DPI or the terminal time point in vector controls, prime and prime + boost groups as well as vector control prime and vector control prime + boost groups clustered similarly, indicating similar overall expression patterns in these respective pairings (Figure 7B). The topmost downregulated (Figure 7C) and upregulated (Figure 7D) mRNAs in specifically vaccinated subjects are depicted. Specifically vaccinated versus vector control samples at late disease (10 DPI or the terminal time point in fatal cases) expressed lower levels of transcripts associated with interferon signaling (e.g., *MX1*, *OASL*, *IFI44*, *GBP1*, *IFIT2*, *IFIT1*) (Figure 7C). Upregulated transcripts were involved in adaptive immunity (e.g., *CD96*, *KLRK1*, *KLRG1*, *KLRF1*, *SH2D1A*) (Figure 7D). Multiple NK cell–associated transcripts were also upregulated in survivors (*KLRC3*, *KLRC2*, *GZMM*), which corroborated our flow cytometry data.

To predict cell-type quantities based on transcriptional signatures, we performed digital cell quantification via nSolver at late disease (Figure 7E). This analysis predicted that survival was associated with increased frequencies of cells involved in adaptive immunity (T helper 1 [Th1] cells, B cells, CD8^+^ T cells, and cytotoxic cells), corresponding to the differential expression analysis. Conversely, lethality regardless of time of vaccination correlated with increased neutrophil frequencies, which was corroborated by our hematology data (Table 1). Lethality was also associated with an increased abundance of exhausted CD8^+^ T cells.

Enrichment of upregulated differentially expressed transcripts (Benjamini-Hochberg–adjusted *P* value < 0.05) in specifically versus nonspecifically vaccinated subjects indicated that survival correlated with activation of pathways involved in memory responses and immunoregulation, e.g., “immune response-regulating signaling,” “adaptive immune response,” “regulation of lymphocyte activation,” and “regulation of B cell activation” (Figure 7F).

## Discussion

In summary, rVSV-ΔG-NiV_B_G is a highly effective and durable vaccine against NiV disease. Several attributes of rVSV-ΔG-NiV_B_G make it an ideal vaccine candidate. rVSV-based vaccines have been tested in hundreds of NHPs with no signs of toxicity (36–43). A vector containing the same backbone (Ervebo) was deemed safe for human use by the US FDA and the European Medicines Agency (29); however, a minor subset of vaccinees developed arthralgia/arthritis, dermatitis, and cutaneous vasculitis in association with viral replication that ultimately resolved (27, 44). The rVSV-ΔG-NiV_B_G vector encodes only one of the two NiV proteins necessary for viral entry and accordingly only undergoes a single round of replication, which may further enhance its safety profile and minimize vaccine-related adverse events such as with Ervebo. Preexisting immunity against the vector backbone is unlikely as VSV seropositivity is low in the general population (45), and Marzi et al. showed that previous vaccination with an rVSV vector did not rescind protection following subsequent immunization with another rVSV-based vaccine (46). Another attractive feature of rVSV-ΔG-NiV_B_G is its inability as a rhabdovirus to reassort or integrate into the host genome, unlike other vectors (47). Finally, rVSV-ΔG-NiV_B_G grows rapidly to high titers, facilitating large-scale manufacturing.

As an individual may not encounter a pathogen for years after immunization, vaccines that provide long-lasting immunity are needed. Our results demonstrate that a single injection of rVSV-ΔG-NiV_B_G provides complete protection in the “gold standard” NHP model, AGMs (15), for at least 1 year after vaccination with no adverse reactions. These results are encouraging as a multidose vaccine regimen requiring several weeks to generate protective immunity is impractical in an outbreak scenario and creates additional logistical issues. A one-shot vaccine approach is preferable and more economical. Remarkably, no overt clinical illness or detectable viral loads were observed in vaccinated subjects, suggesting that rVSV-ΔG-NiV_B_G may induce sterilizing immunity. Vector control AGMs instead exhibited high viral loads and common NiV disease features such as anorexia, hematological and serum biochemistry changes, depression, respiratory distress, and neurological deficits. These animals succumbed within the typical time-to-death for this experimental model (7–9 DPI) (15).

Although the precise mechanisms of rVSV-mediated immunity against NiV disease are not yet understood, we show that rVSV-ΔG-NiV_B_G potently activates humoral responses. Similarly, an experimental immune cell depletion study in NHPs revealed that antibodies are essential for Ervebo protection against Ebola virus disease (43). CD4^+^ T cell depletion during vaccination, but not during Ebola virus challenge, prevented formation of glycoprotein-specific IgG and abrogated protection. These results suggest that CD4^+^ T cell helper (i.e., B cell maturation and antibody isotype class switching) versus effector functions are pivotal for Ervebo-mediated protection. CD8^+^ T cells were instead dispensable against the pathogen. However, mechanisms of protection may differ for these two vaccine platforms; therefore, similar depletion studies are needed for NiV infection.

In this study, G-specific IgG titers in rVSV-ΔG-NiV_B_G–vaccinated AGMs correlated with respiratory health, indicating that the presence of this immune constituent may reliably predict protection. Vaccination induced stable and moderate to robust circulating neutralizing and G-specific IgG titers, but only low IgM levels were generated. In both specifically vaccinated groups, binding and neutralizing antibody levels waned half a year after the initial prime dose, but anamnestic IgG (1:12,800 to 1:819,200) and neutralizing (1:640 to 1:10,240) titers were generated following NiV_B_ exposure that peaked during convalescence. In contrast, nonspecific control antibody levels remained low or below the limit of detection for our assay throughout the entire study. A booster dose transiently augmented antibody responses during the vaccination phase but did not offer additional efficacy and by 5 months matched levels elicited by a single dose of rVSV-ΔG-NiV_B_G. Therefore, a booster does not appear to be essential for at least 1 year but may provide benefit for longer intervals.

Cellular responses induced by rVSV-ΔG-NiV_B_G vaccination may play a supportive role in conferring resistance against NiV disease. The exact cell subsets contributing to cellular immunity against NiV disease remain largely unexplored, yet other studies have demonstrated the involvement of NK cells in rVSV-mediated protection against NiV (26), Lassa virus (33), Ebola virus (48), and Marburg virus (34, 35) in NHPs. Our flow cytometry results show increased frequencies of total, cytotoxic, and IFN-γ–secreting NK cells in PBMC samples from specifically immunized subjects in the present study, in addition to the expression of NK cell–associated transcripts. In humans, the Ervebo vaccine was reported to modulate CD56^+^ NK cell counts and the expression of various NK surface receptors such as NKG2D, NKp30, and killer immunoglobulin-like receptors shortly after vaccination. A systems vaccinology approach also demonstrated that the total frequency of CD56^+^ NK cell count and CXCR6 expression on NK cells correlated with the antibody response to Ervebo in healthy adults (49). Thus, NK cells may contribute to rVSV protection in myriad ways. Other cellular effectors such as helper and effector T cells may also participate in host defense. Specific IFN-γ ImmunoSpot assay responses directed at the NiV_B_G, albeit modest, were observed in 5 of 6 prime-only and 4 of 5 prime-boosted subjects over the course of the study. Digital cell quantitation via whole blood transcriptomics corroborated a predicted increase in circulating Th1 and CD8^+^ T cell frequencies in specifically immunized AGMs. Moreover, we detected a higher abundance of total CD4^+^ T and CD8^+^ T cell counts, and higher antigen-specific T cell polyfunctionality, in rVSV-ΔG-NiV_B_G–vaccinated subjects.

One caveat of this study is that only bulk transcriptomics was performed. Although digital cell quantitation and flow cytometry were executed, single-cell sequencing and deep immunophenotyping will better elucidate the role of specific cell subsets in rVSV-ΔG-NiV_B_G–mediated immunity. For example, certain transcriptional signatures indicate that innate lymphoid cells might be involved in protection. Although single-cell sequencing may offer a deeper insight into the immune response of specific cell types, bulk transcriptomics offers a broader view of the immune response in the vaccinated animals.

In conclusion, the rVSV-ΔG-NiV_B_G vaccine provides durable protection against NiV disease by inducing long-lived adaptive responses. This vaccine will be a useful tool in curtailing future outbreaks of the virus, as near-annual cases are still reported in India and Bangladesh with high mortality rates. Breakthrough infections have been commonly experienced following COVID-19 vaccination partly because of waning immunity (50), which may fuel vaccine hesitancy. COVID-19 vaccines so far have also failed to provide sterilizing protection. We demonstrate that rVSV-ΔG-NiV_B_G provides durable immunity with no detectable NiV replication, which may bolster public confidence in the vaccine. Future work will include manufacturing clinical-grade vaccine lots, determining optimal dosing regimens, and improving temperature stability, as currently rVSV-ΔG-NiV_B_G requires –80°C long-term storage. Importantly, generation of stable cell lines expressing the VSV glycoprotein will be critical for large-scale manufacturing. Evaluation of efficacy at longer gaps between vaccination and challenge should also be conducted to inform public health policy decision making, e.g., timing of booster vaccinations and effective response to a pandemic.

## Methods

Further information can be found in Supplemental Methods.

### Study design.

To test the vaccine durability of rVSV-ΔG-NiV_B_G, AGMs were immunized with 1 or 2 doses of either a vector control or rVSV-ΔG-NiV_B_G vaccine. A power analysis was conducted to determine the sample size necessary to achieve a reliable measurement of effect for each animal cohort. Sampling time points and the final endpoint of the study (35 DPI) were established prospectively. Scoring criteria for clinical assessment of animal health were also predetermined. Study results were from a single animal experiment. Eighteen AGMs were randomized into 4 groups: prime only (*n* = 6), prime + boost (*n* = 6), vector control prime (*n* = 3), and vector control prime + boost (*n* = 3). A single subject in the prime + boost group was euthanized within the vaccination phase owing to issues deemed unrelated to the study or vaccination, resulting in a final total of 5 subjects for that group. No data were excluded for any other subject. An outlier (single replicate) in the ELISPOT data was excluded because of a known experimental error; this is reported in the figure legend. For all assays, results were from a single experiment with the average of duplicates reported for each sample. All subjects were included for our analyses. The project administrator was not blinded to the allocation sequence. Animal caretakers and investigators conducting the experiments were blinded to the allocation sequence, conduct of the experiment, and outcome assessment. Investigators who assessed, measured, or quantified the results were not blinded to the intervention for data analysis.

### Characterization of rVSV-ΔG-NiV_B_G vaccine.

The rVSV-ΔG-NiV_B_G vaccine was recovered, sequenced, and characterized as described previously (26). The vaccine stocks tested negative for mycoplasma and endotoxin contamination.

### Challenge virus.

The NiV_B_ challenge material used in the study (200401066) originated from a fatal human case during an outbreak in Rajbari, Bangladesh, in 2004. The challenge material was passaged twice onto Vero E6 cells (ATCC CRL-1586), and supernatants were collected and stored at –80°C as approximately 1-mL aliquots. Four distinct mutations of sufficient frequency were found between the P2 stock of NiV_B_ and the reference genome (GenBank AY988601.1). One mutation was noncoding whereas the remaining mutations encode for 3 single amino acid changes: 1 in the M protein and 2 in the F protein (7). No detectable mycoplasma or endotoxin was present in our virus seed stock (<0.5 endotoxin units/mL).

### NHP vaccination and challenge.

Seventeen healthy, adult AGMs (8 males and 9 females) from St. Kitts (*Chlorocebus aethiops*; Worldwide Primates) were randomized into 4 groups: prime only (*n* = 6), prime + boost (*n* = 5), vector control prime (*n* = 3), and vector control prime + boost (*n* = 3). The 6 experimental animals were specifically vaccinated by intramuscular (i.m.) injection of 1 × 10^7^ PFU of rVSV-ΔG-NiV_B_G, and control animals were vaccinated by i.m. injection of 1 × 10^7^ PFU of the nonspecific vector. One year after prime vaccination, all AGMs were exposed to 5 × 10^3^ PFU of NiV_B_ intranasally using the LMA Mucosal Atomization Device as previously described (32).

All animals for both studies were given physical examinations, and blood was collected before vaccination (day 0) and on days 4, 7, 10, 14/15, 21, 28, and 35 after virus challenge. The AGMs were monitored daily and scored for disease progression with an internal NiV humane endpoint scoring sheet approved by the University of Texas Medical Branch (UTMB) IACUC. Scoring criteria were based on parameters such as respiration (0 to 9), appetite (0 to 2), activity/appearance (0 to 9), and neurological signs (0 to 9). A score greater than or equal to 9 met euthanasia criteria. UTMB facilities used in this work are accredited by the Association for Assessment and Accreditation of Laboratory Animal Care International and adhere to principles specified in the 8th edition of the Guide for the Care and Use of Laboratory Animals, National Research Council (National Academies Press, 2011). The scoring changes measured from baseline included posture and activity level, attitude and behavior, food intake, respiration, and central nervous system abnormalities.

### Blood processing and PBMC isolation.

Blood was collected by femoral venipuncture into EDTA, heparin, and clot-activating vacutainer tubes (BD Biosciences). The EDTA plasma and serum tubes were centrifuged at approximately 1,300*g* at 4°C for 10 minutes; afterward, the upper layer was collected. For isolation of PBMCs, heparin-treated blood and the spun EDTA pellet were diluted with PBS and carefully layered onto a Histopaque cushion within Accuspin tubes (Sigma-Aldrich). The tubes were centrifuged at approximately 800*g* room temperature for 15 minutes, and the resulting buffy coat was collected. Cells were washed once in R10 (RPMI medium [Gibco] supplemented with 10% FBS, 100 U/mL penicillin, 100 g/mL streptomycin solution, and 1% l-glutamine) and treated briefly with ACK lysing buffer (Gibco) to remove any contaminating erythrocytes. PBMCs were then centrifuged at approximately 250*g* for 10 minutes to eliminate residual thrombocytes, washed twice with R10 medium, and enumerated with a TC20 Automated Cell Counter (Bio-Rad). Cells were cryopreserved in 10% DMSO in FBS. Before flow cytometry, cryopreserved PBMCs were thawed rapidly in a 37°C water bath (BD Biosciences).

### Hematology and serum biochemistry.

Total red blood cell counts, white blood cell counts, white blood cell differentials, platelet counts, hematocrit values, total hemoglobin concentrations, mean cell volumes, mean corpuscular volumes, and mean corpuscular hemoglobin concentrations were analyzed from blood collected in tubes containing EDTA using a Vetscan HM5 laser-based hematological analyzer (Zoetis). Serum samples were tested for concentrations of albumin, amylase, alanine aminotransferase, aspartate transaminase, alkaline phosphatase, γ-glutamyltransferase, blood urea nitrogen, creatinine, C-reactive protein, calcium, glucose, total protein, and uric acid using a Piccolo point-of-care analyzer and Biochemistry Panel Plus analyzer discs (Abaxis).

### RNA isolation from NiV_B_-infected AGMs.

On the specified procedure days, 100 μL of blood was added to 600 μL of AVL viral lysis buffer (Qiagen) for RNA extraction. For tissues, approximately 100 mg of sample was stored in 1 mL RNAlater (Qiagen) for 7 days for stabilization. RNAlater was removed and tissues were homogenized in 600 μL RLT buffer (Qiagen) in a 2 mL cryovial using a Tissue Lyser (Qiagen) and ceramic beads. The tissues sampled included axillary, inguinal, mandibular, and mesenteric lymph nodes; upper, middle, and lower lobes of both left and right lungs; spleen; liver; kidney; adrenal gland; frontal cortex of brain; brainstem; cervical spinal cord; submandibular salivary gland; tonsil; heart; duodenum; pancreas; ileocecal junction; transverse colon; urinary bladder; ovary or testis; uterus or prostate; nasal mucosa; conjunctiva; and eye. All blood samples were inactivated in AVL viral lysis buffer, and tissue samples were homogenized and inactivated in RLT buffer before removal from the biosafety level 4 (BSL-4) laboratory. Subsequently, RNA was isolated from blood using the QIAamp viral RNA kit (Qiagen), and from tissues using the RNeasy Mini Kit (Qiagen), according to the vendor instructions supplied with each kit.

### Quantification of viral load.

Viral loads of RNA from blood or tissues were measured using reverse transcriptase quantitative PCR (RT-qPCR) and primers/probe targeting the nucleoprotein (N) gene and intergenic region between N and phosphoprotein (P) of NiV_B_. Probe sequences were 6FAM-5′-CGTCACACATCAGCTCTGACAA-3′-6TAMRA for NiV_B_ (Life Technologies). Threshold cycle values representing viral genomes were analyzed with CFX Manager software (Bio-Rad); the data are displayed as genome equivalents (GEq). To create the GEq standard, RNA from viral stocks was extracted, and the number of genomes present was calculated using Avogadro’s number and the molecular weight of the genome.

Virus titration was performed by plaque assay using Vero 76 cells (ATCC CRL-1587) from all plasma samples. Briefly, increasing 10-fold dilutions of the samples were adsorbed to Vero 76 cell monolayers in duplicate wells (200 μL/well) and overlaid with 0.8% agarose in 1× minimum essential medium (MEM) with 5% FBS and 1% penicillin/streptomycin. After a 2- to 3-day incubation at 37°C and 5% CO_2_, neutral red stain was added, and plaques were counted after an additional 24-hour incubation. The limit of detection for this assay is 25 PFU/mL.

### ELISA.

Sera collected at the indicated time points were tested for total anti-NiV IgG and IgM antibodies by ELISA using monkey species–specific kits (Alpha Diagnostic International, NIV-015 and NIV-020) following the vendor recommendations.

### Plaque reduction neutralization test.

Neutralization titers were calculated by determining the dilution of serum that reduced 50% of plaques (PRNT_50_). A standard 100 PFU amount of NiV_B_ was incubated with 2-fold serial dilutions of serum samples in DMEM for 1 hour. The virus-serum mixture was then used to inoculate Vero 76 cells (ATCC CRL-1587) for 30 minutes. Cells were overlaid with 2× MEM agar medium and incubated for 2–3 days, and plaques were counted after 24 hours of 5% neutral red staining.

### RNA sample preparation for transcriptomic analyses.

NHPV2_Immunology reporter and capture probe sets (Nanostring Technologies) were hybridized with approximately 3 μL of blood RNA at 65°C for approximately 24 hours as previously described (51). Following the hybridization, the RNA–probe set complexes were loaded into an nCounter microfluidics cartridge and assayed on a NanoString nCounter SPRINT Profiler. To estimate the abundance of each of the 769 unique mRNA immune-related targets included in the NHPV2_Immunology panel, fluorescent reporter barcodes were imaged and counted for each sample lane.

### Bioinformatics.

The nCounter recap compressed structured scan data (RCC) files were imported into NanoString nSolver 4.0 software. All samples met the integrated quality control criteria. To compensate for varying RNA inputs, housekeeping genes and spiked-in positive and negative controls were incorporated to normalize raw counts. The data were analyzed using the NanoString nSolver Advanced Analysis 2.0 package to generate principal component analysis figures and differential expression heatmaps. Normalized data (log fold change values and Benjamini-Hochberg–adjusted *P* values) for each sample group were exported as a.CSV file (Microsoft Excel Office for Mac v14.1.0). MetaScape (52) was used for pathway analysis of differentially expressed transcripts (Benjamini-Hochberg–adjusted *P* value < 0.05 for prime vs. vector control group) using human annotations and the default settings (3 minimum overlap, 1.5 minimum enrichment). GraphPad Prism version 9 was used to produce heatmaps. Human annotations were added for each respective gene to perform immune cell profiling and generate cell-type plots within nSolver.

### Histology.

Tissue sections were deparaffinized and rehydrated through xylene and graded ethanol washes. Slides went through heat antigen retrieval in a steamer at 95°C for 20 minutes in Sigma Citrate Buffer, pH 6.0, 10× (Sigma-Aldrich). To block endogenous peroxidase activity, slides were treated with 3% hydrogen peroxide and rinsed in distilled water. The tissue sections were processed for IHC using the Thermo Autostainer 360 (Thermo Fisher Scientific). Sequential 15-minute incubations with avidin D and biotin solutions (Vector Laboratories, SP-2001) were performed to block endogenous biotin reactivity. Specific anti-NiV immunoreactivity was detected using an anti-NiV m102.4 human monoclonal antibody (53) at a 1:4,000 dilution for 60 minutes. Secondary antibody was biotinylated goat anti-rabbit IgG (Vector Laboratories, BA-1000) at 1:200 for 30 minutes followed by Vector Horseradish Peroxidase Streptavidin (ready-to-use; Vector Laboratories, SA-5704) for 30 minutes. Slides were developed with Dako DAB chromogen (Dako, K3468) for 5 minutes and counterstained with hematoxylin for 45 seconds.

### ELISPOT.

To analyze cellular responses, NHP PBMCs were rapidly thawed in a water bath at 37°C and resuspended in prewarmed complete RPMI 1640 medium with 10% FBS, 1% GlutaMAX (Thermo Fisher Scientific), and 1% penicillin/streptomycin (Thermo Fisher Scientific). Cells were rested overnight at 37°C and 5% CO_2_. After the resting period, PBMCs were counted and either left unstimulated or stimulated for approximately 24 hours at 37°C and 5% CO_2_ with either lectin (Sigma-Aldrich) from *Phytolacca americana* (PWM) or a custom NiV_B_ G peptide pool (GenScript) spanning the length of G. The NiV_B_ G peptide pool contained 148 × 15-mer peptides with 11 amino acid overlaps. The lyophilized pool was prepared in DMSO and used at a final concentration of 2 μg/mL, whereas unstimulated cells contained 0.2% DMSO by volume. As a positive stimulation control, PBMCs were stimulated with PWM at a final concentration of 0.5 μg/mL. For ELISPOT analysis, samples were stained using single-color primate IFN-γ kits (Mabtech AB) according to the manufacturer’s recommendations. PBMCs were plated in duplicate at 2.5 × 10^5^ cells per well in a 96-well plate coated with NHP IFN-γ capture antibody. After an approximately 24-hour incubation at 37°C and 5% CO_2_, ELISPOT plates were air-dried and imaged using an ImmunoSpot S6 UNIVERSAL Analyzer (Cellular Technology Ltd.). Reported values were calculated by subtraction of the number of spot-forming cells (SFCs) in each unstimulated sample from its respective stimulated counterpart at the corresponding DPI.

### Flow cytometry.

To examine the polyfunctionality and frequency of NK and T cell populations, we performed intracellular cytokine staining. In the presence of anti-CD28 (BioLegend clone CD28.2; RRID:AB_314304), CD49d (BioLegend clone 9F10; RRID:AB_2130039), and CD107 (BioLegend clone H4A3; RRID:AB_1279055; APC) antibodies, PBMCs were stimulated for 6 hours with a DMSO negative control, a PWM (0.5 μg/mL) positive control, or 2 μg/mL of an overlapping NiV G peptide pool (148 × 15-mers overlapping by 11 amino acids; custom-made at GenScript). Brefeldin A protein transport inhibitor (Sigma-Aldrich, catalog B6542) was added 4 hours before surface staining of CD3 (BD Biosciences clone SP34-2; RRID:AB_396484; FITC), CD4 (BD Biosciences clone L200; RRID:AB_394488; PerCP/Cy5.5), and CD8α (BD Biosciences clone SK1; RRID:AB_1953244; PE). Two micrograms per milliliter DNase (Invitrogen, catalog AM2224) and rhesus Fc receptor binding inhibitor (Thermo Fisher Scientific; RRID:AB_2572937) were added to reduce clumping and nonspecific binding. Cells were subsequently washed in BD Biosciences staining buffer and permeabilized using a Foxp3/Transcription Factor Staining Buffer kit (Tonbo Biosciences, catalog TNB-0607) as suggested by the manufacturer. After permeabilization, we stained for the intracellular markers TNF-α (BD Biosciences clone mAB11; RRID:AB_2204079; PE/Cy7), IFN-γ (BioLegend clone B27; RRID:AB_2801098; BV421), and IL-2 (BioLegend clone MQ1-17H12; RRID:AB_2562855; APC/Cy7).

Approximately 200,000 events were collected on a FACSCanto II cytometer (BD Biosciences) for each sample using BD FACSDiva software. Data were analyzed with FlowJo version 10 software (Tree Star). Live versus dead cells were distinguished by BV510 fixable viability dye (BD Biosciences catalog 564406; RRID:AB_2869572). Compensation was calculated using BD CompBeads (BD Biosciences; RRID:AB_1727537) or single-color-stained and fixed cells. Flow Cytometry Standard (FCS) files were imported into NIH Simplified Presentation of Incredibly Complex Evaluations (SPICE) (54) for polyfunctionality analysis. Reported values were calculated by subtraction of the subset frequencies in each unstimulated sample from their respective peptide-stimulated counterparts at the corresponding DPI.

### Statistics.

The survival of prime-only (specific vs. control) and prime-boosted (specific vs. control) groups was compared using a log-rank test. Statistical tests were performed using Prism 9 (GraphPad). All data are derived from a single animal experiment. Statistics were derived from average values from the following 4 cohorts: prime only (*n* = 6), prime + boost (*n* = 5), vector control prime (*n* = 3), and vector control prime + boost (*n* = 3). Statistics for all figures were calculated from individual animal data values rather than technical replicates. For experiments with technical replicates (for example, duplicate RT-qPCR reactions/wells), only the mean was used to calculate statistical significance. A 2-way ANOVA with Tukey’s multiple-comparison test was used to determine statistical significance between prime-only (specific vs. control) and prime-boosted (specific vs. control) groups for viral loads, humoral responses, and cellular responses. For cytokine bead array measurements, the results of fold change calculations and ANOVA with Tukey’s post hoc test were calculated using the rstatix (v0.7.0) package (GNU Guix). A multiple-hypothesis Benjamini-Hochberg–corrected *P* value less than 0.05 was deemed significant for transcriptomic analyses.

Representative photomicrographs were qualitatively considered to display lesions that were nominally or ordinally measured by masking of the pathologist after examination and ranking of lesions to satisfy study objectives. Additionally, a thorough examination of multiple slides of target tissues (for example, 18 slides of lung) multiple times (up to 3 times per tissue) was performed in a timely manner to maintain interpretation consistency.

### Study approval.

Monkeys were handled in animal BSL-2 and BSL-4 containment in the Galveston National Laboratory at the University of Texas Medical Branch (UTMB), Galveston, Texas. This facility is assured by the Office of Laboratory Welfare and fully accredited by the Association for Assessment and Accreditation of Laboratory Animal Care International. All research was approved by the UTMB IACUC and complied with the Animal Welfare Act and other federal statutes and regulations pertaining to animal experimentation. Provisions were taken to prevent, ameliorate, and minimize pain and distress of the animals. Animals were monitored by an attending veterinarian and scored at least twice daily for food intake, responsiveness, weakness, recumbency, labored breathing, diarrhea, edema, dehydration, and the presence of coagulopathies. Animals meeting humane endpoint scoring criteria were promptly euthanized with a pentobarbital solution.

## Author contributions

RWC and TWG conceived and designed the animal challenge experiments. CW, DJD, JBG, RWC, and TWG performed the animal procedures. CW, DJD, ANP, RWC, and TWG conducted clinical observations. KNA and VB performed the clinical pathology. KNA performed the PCR assays. KNA and RO performed the ELISAs. VB performed the NiV infectivity and neutralization assays. CW, ANP, and SLF prepared PBMC separations. CW and ACF performed ELISPOT assays. ACF performed the LegendPlex assay. CW performed the NanoString assays. NSD performed the IHC assays. KAF performed gross pathological, histological, and immunohistochemical analysis of the data. All authors analyzed the data. CW authored the paper. ANP, KAF, RWC, and TWG edited the paper. All authors had access to the data and approved the final version of the manuscript.

## Supplementary Material

Supplemental data

## Figures and Tables

**Table 1:**
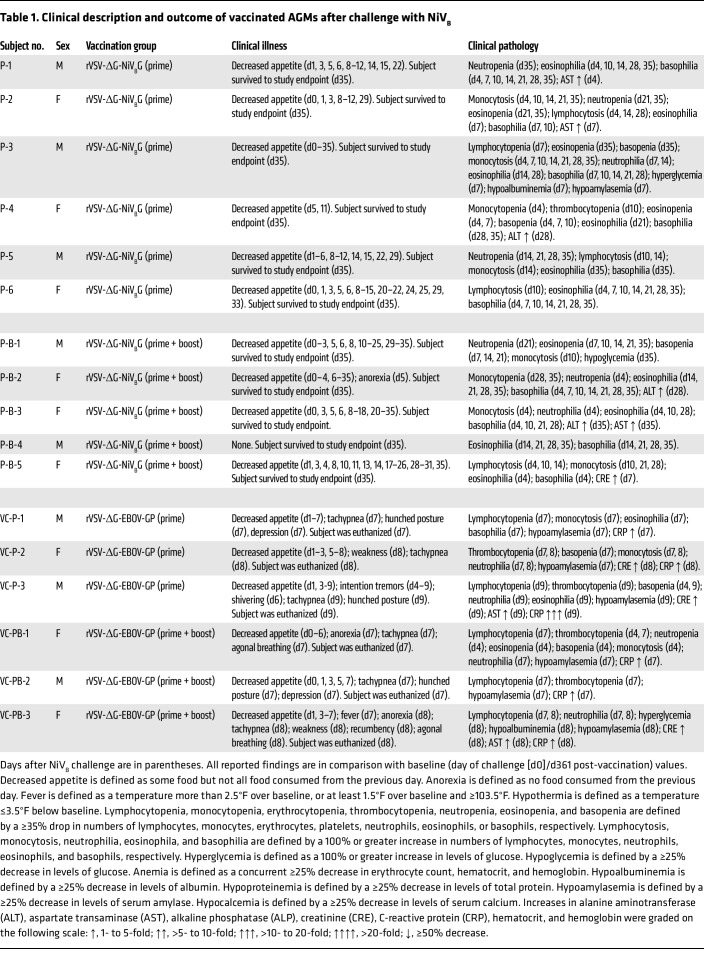
Clinical description and outcome of vaccinated AGMs after challenge with NiV_B_

## Version 1 11/29/2022

In-Press Preview

## Version 2 02/01/2023

Electronic publication
