# Supplementary material for: Recombinant vesicular stomatitis virus–vectored vaccine induces long-lasting immunity against Nipah virus disease
Source: J Clin Invest. 2023 Feb 1;133(3):e164946. doi: 10.1172/JCI164946 (PMC9888376; doi:10.1172/JCI164946)
Supplement: Supplemental data [file jci-133-164946-s074.pdf]

Supplementary Materials

Materials and Methods
Supplemental Figures 1 and 2
Supplemental Table 1
Supplemental References

Materials and Methods

Bead-based multiplex assay

Plasma analytes were measured by flow cytometry using Biolegend LegendPlex™ assays and a FACS Canto II cytometer (Becton Dickson). Samples were processed in duplicate using a BioLegend Nonhuman Primate Inflammation 13-plex kit (1:4 dilution) according to the manufacturer's instructions. To ensure consistency among plates, standards were mixed in batch and aliquoted across all plates. Optional wash steps were incorporated to reduce background. Fold change calculations were plotted using the package Pheatmap v1.0.12 in R. Results of fold change calculations and ANOVA with Tukey post-hoc tests were calculated and plotted using ggplot2 (v3.3.5) (ref. S1), ggbreak (v0.0.8) (ref. S2), viridis (v0.6.2) (ref. S3), and rstatix (v0.7.0) packages.

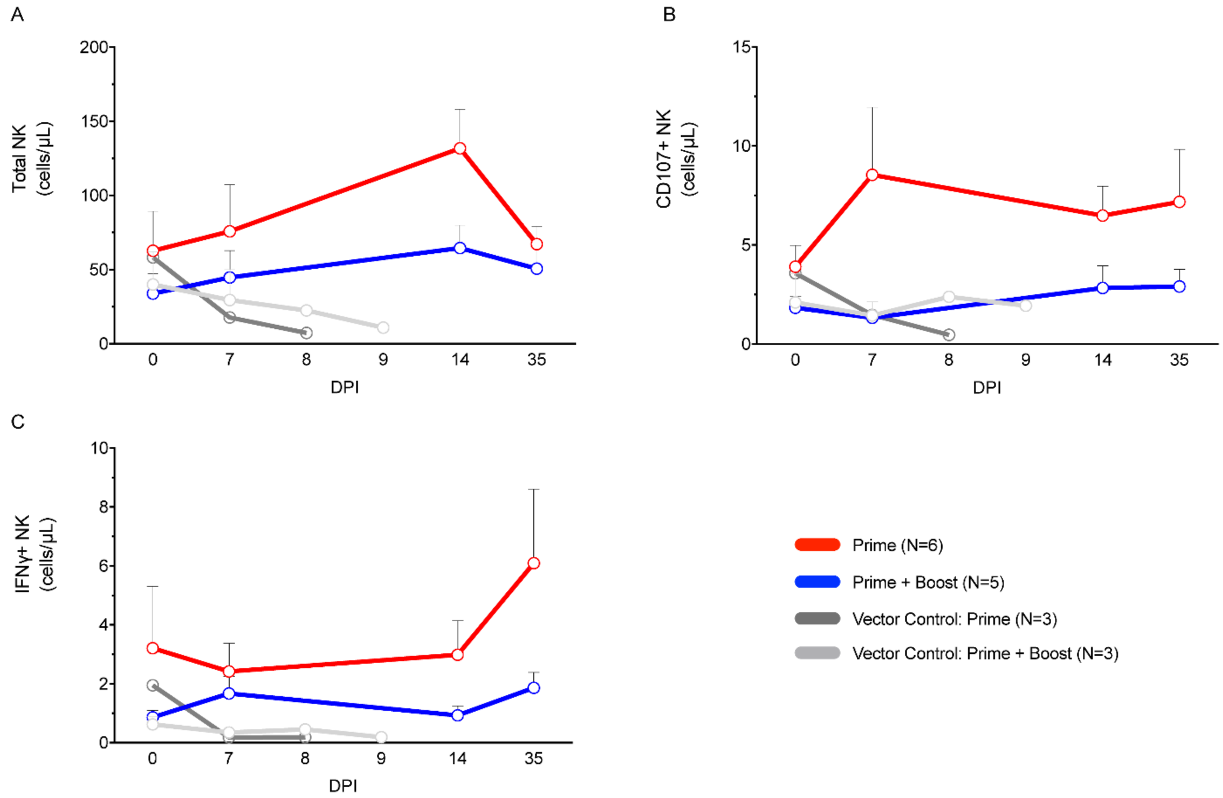

Supplemental Figure 1. NK cell responses in vaccinated AGM PBMCs after challenge with NiV$_B$. Line graphs depicting the average absolute numbers of total (CD3-CD8α+) (**A**), degranulating (CD3-CD8α+CD107+) (**B**), and IFNγ-secreting (CD3-CD8α+ IFNγ+) (**C**) NK cells in PBMC from each AGM group. Each group is denoted by the following: prime only (rVSV-ΔG-NiV$_B$G N=6; red lines), prime + boost (rVSV-ΔG-NiV$_B$G N=5; blue lines), vector control prime (rVSV-ΔG-EBOV-GP N=3; dark gray lines), and vector control prime + boost (rVSV-ΔG-EBOV-GP N=3; light gray lines). Error bars represent the SEM for each group for all panels. Abbreviations: NK, natural killer; AGM, African green monkey; PBMC, peripheral blood mononuclear cells; NiV$_B$, Nipah virus Bangladesh strain; DPI, days post infection; rVSV, recombinant vesicular stomatitis virus; G, NiV$_B$ glycoprotein; EBOV-GP, Ebola virus glycoprotein.

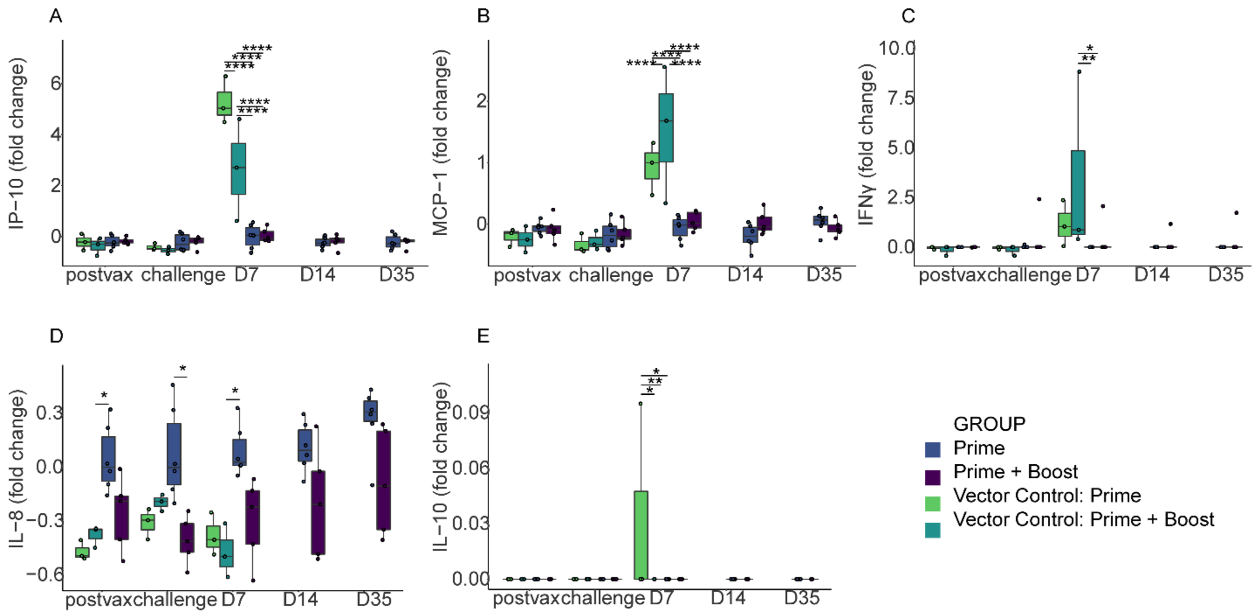

Supplemental Figure 2. Expression of soluble mediators in the plasma of immunized AGMs following NiV$_B$ exposure. Boxplots displaying group fold-change values for IP-10 (**A**), MCP-1 (**B**), IFNγ (**C**), IL-8 (**D**), and IL-10 (**E**) in in the plasma of vaccinated AGMs following NiV$_B$ challenge. Fold change depicted was calculated from a pre-challenge baseline. Each group is denoted by box color: prime only (rVSV-ΔG-NiV$_B$G N=6; blue), prime + boost (rVSV-ΔG-NiV$_B$G N=5; purple), vector control prime (rVSV-ΔG-EBOV-GP N=3; green), and vector control prime + boost (rVSV-ΔG-EBOV-GP N=3; teal). For all panels, boxes whiskers denote range of data. No outliers were excluded. Abbreviations: postvax, post-immunization; D, day post-challenge; AGM, African green monkey; NiV$_B$, Nipah virus Bangladesh strain; IP-10, interferon gamma-induced protein 10; MCP-1, monocyte chemoattractant protein-1; IFNγ, interferon gamma; IL-8, interleukin-8; IL-10, interleukin-10; DPI, days post infection; rVSV, recombinant vesicular stomatitis virus; G, NiV$_B$ glycoprotein; EBOV-GP, Ebola virus glycoprotein. Two-way ANOVA with Tukey's multiple comparisons test. p-value < 0.0332 (*), 0.0021 (**), 0.0002 (***), 0.0001 (****).

Supplemental Table 1. Clinical pathology of AGMs following vaccination

Subject No.	Sex	Vaccination Group	Clinical pathology
P-1	M	rVSV-ΔG-NiV$_{B}$G (Prime)	Lymphocytopenia (d294, 328); neutropenia (d84, 139); eosinopenia (d28, 84, 139, 164, 195, 221); basopenia (d28, 84, 139, 164, 195); monocytosis (d84, 195, 259); neutrophilia (d259); eosinophilia (d259); basophilia (d259); hypoglycemia (d0); hypoamylasemia (d195).
P-2	F	rVSV-ΔG-NiV$_{B}$G (Prime)	Lymphocytopenia (d139, 195, 221, 259, 294, 329); monocytopenia (d139, 294); thrombocytopenia (d112); neutropenia (d0, 10, 28, 56, 84, 112, 139, 164, 195, 221, 294, 329); eosinopenia (d0, 10, 28, 56, 84, 112, 139, 164, 195, 221, 294, 329); basopenia (d0, 10, 28, 56, 84, 112, 139, 164, 195, 221, 259, 294, 329); monocytosis (d164, d221); hypoamylasemia (d221); ALT ↑ (d294, 329); GGT ↑ (d329).
P-3	M	rVSV-ΔG-NiV$_{B}$G (Prime)	Monocytopenia (d84, 195); thrombocytopenia (d259); neutropenia (d10, 28, 84, 112, 139, 195, 221, 294, 329); eosinopenia (d0, 10, 28, 84, 112, 139, 164, 195, 221, 294, 329); basopenia (d0, 10, 28, 84, 112, 139, 164, 195, 294, 329); monocytosis (d10, 28, 56, 112, 221, 329); ALT ↑ (d28, 329).
P-4	F	rVSV-ΔG-NiV$_{B}$G (Prime)	Lymphocytopenia (d259, 329); thrombocytopenia (d56, 84); monocytopenia (d84, 164, 259, 294, 329); neutropenia (d164); eosinopenia (d112, 164, 221); basopenia (d112, 164); eosinophilia (d56); ALT ↑ (d28, 112, 164, 195, 294, 329).
P-5	M	rVSV-ΔG-NiV$_{B}$G (Prime)	Lymphocytopenia (d28); monocytopenia (d56, 164, 221, 259, 294); thrombocytopenia (d28); neutropenia (d195, 221, 294); eosinopenia (d0, 28, 84, 195, 221, 259, 294); thrombocytosis (d0, 10, 84, 112, 139, 164, 195, 221, 259, 294, 329); hypoalbuminemia (d112); CRE ↑ (d0, 56, 84, 139, 329).
P-6	F	rVSV-ΔG-NiV$_{B}$G (Prime)	Lymphocytopenia (d0, 221, 329); monocytopenia (d0, 329); thrombocytopenia (d28, 56, 164, 195, 259, 329); neutropenia (d10, 112, 139); eosinopenia (d0, 10, 84, 139, 164, 329); basopenia (d56, 84, 139, 164, 329); hypoamylasemia (d329); ALT ↑ (d164, 195, 221, 259, 294, 329).
PB-1	M	rVSV-ΔG-NiV$_{B}$G (Prime + Boost)	Thromobocytopenia (d56); monocytosis (d10, 28, 112, 195, 329); neutrophilia (d84, 259); eosinophilia (d56, 84, 259); basophilia (d84, 259); hypoglycemia (d259); AST ↑ (d10).
PB-2	F	rVSV-ΔG-NiV$_{B}$G (Prime + Boost)	Lymphocytopenia (d259); monocytopenia (d10, 139, 164, 195); thrombocytopenia (d10, 294); neutropenia (d112, 164, 195, 221); eosinopenia (d112, 164, 195, 221); basopenia (d112, 164, 221); lymphocytosis (d84); monocytosis (d84); hypoglycemia (d294, 329); ALT ↑ (d56, 84, 112, 164, 195, 221, 259, 294).
PB-3	F	rVSV-ΔG-NiV$_{B}$G (Prime + Boost)	Thrombocytopenia (d294); neutrophilia (d10, 164, 221, 259, 294); eosinophilia (d0, 10, 139, 164, 195 221, 259, 294, 329); basopenia (d0, 10, 164, 195, 221, 294); monocytosis (d10, 28, 56, 84, 112, 139, 164, 195, 221, 259, 294, 329); hypoglycemia (d221); ALT ↑ (d56, 84, 195, 294); AST ↑ (d56, 84).
PB-4	F	rVSV-ΔG-NiV$_{B}$G (Prime + Boost)	Monocytopenia (d0, 195, 259, 294); thrombocytopenia (d259, 294); monocytosis (d10, 84, 164, 221); neutrophilia (d10, 84, 139, 164, 195, 221, 259, 294); eosinopenia (d0, 10, 56, 84, 112, 139, 164, 195, 221, 259); basopenia (d112, 139, 164, 221, 259); hypoglycemia (d221); ALT ↑ (d84); AST ↑↑ (d84).
PB-5	M	rVSV-ΔG-NiV$_{B}$G (Prime + Boost)	Lymphocytopenia (d139, 294, 329); monocytopenia (d195); eosinopenia (d164, 195); basopenia (d0, 10, 28, 56, 84, 112, 139, 164, 195, 221, 259, 294, 329); monocytosis (d10, 164, 221, 329); eosinophilia (d112, 221, 259); AST ↑ (d112, 221); GGT ↑ (d195).
VC-P-1	M	rVSV-ΔG-EBOV-GP (Prime)	Lymphocytopenia (d259, 294); thrombocytopenia (d10, 28, 56, 139, 164, 195, 329); neutropenia (d195); eosinopenia (d0, 10, 28, 84, 112, 164, 195, 221, 259, 294, 329); basopenia (d0, 10, 164, 195, 329); monocytosis (d56, 84, 139, 221, 259, 294, 329); neutropenia (d56, 139); ALT ↑ (d28, 56); AST ↑ (d28, 56).
VC-P-2	F	rVSV-ΔG-EBOV-GP (Prime)	Thrombocytosis (d259); monocytosis (d0, 294); neutropenia (d112, 195, 221); eosinopenia (d0, 10, 139, 164, 195, 221, 329); basopenia (d0, 10, 164, 195, 221, 329); monocytosis (d84, 329); neutrophilia (d84); eosinophilia (d84); basophilia (d84); hypoglycemia (d0, 221, 259); hypoamylasemia (d221, 294, 329); CRE ↑ (d259).
VC-P-3	M	rVSV-ΔG-EBOV-GP (Prime)	Lymphocytopenia (d56, 139, 164, 221, 259, 294, 329); neutropenia (d139, 164, 195, 294, 329); eosinopenia (d0, 10, 28, 84, 139, 164, 195, 221, 294, 329); basopenia (d0, 84, 139, 164, 195, 221, 259, 294, 329); monocytosis (d0, 10, 28, 112, 164, 221, 329); ALT ↑ (d294); AST ↑ (d56).
VC-PB-1	F	rVSV-ΔG-EBOV-GP (Prime + Boost)	Monocytopenia (d0, 28, 56, 112, 139, 164, 259); thrombocytopenia (d139); neutropenia (d0, 10, 28, 56, 112, 139, 164, 195, 221, 294, 329); eosinopenia (d0, 10, 28, 56, 112, 139, 164, 195, 221, 259, 294, 329); basopenia (d0, 10, 28, 56, 112, 139, 164, 195, 221, 294); lymphocytosis (d0, 112, 164, 195, 221, 329); hyperglycemia (d329).

VC-PB-2	M	rVSV-ΔG-EBOV-GP (Prime + Boost)	Monocytopenia (d10, 28, 84, 112, 139, 164, 259, 294, 329); neutropenia (d0, 195, 221, 259, 294, 329); eosinopenia (d84); basopenia (d84, 294, 329); lymphocytosis (d195); eosinophilia (d56, 112, 329); basophilia (d56, 112); hypoglycemia (d0); hypoalbuminemia (d0); hypoamylasemia (d0); ALT ↑ (d56, 84, 164, 294, 329).
VC-PB-3	F	rVSV-ΔG-EBOV-GP (Prime + Boost)	Monocytopenia (d0, 10, 56, 139, 221, 259, 294); thrombocytopenia (d56, 221, 294); neutropenia (d0, 139, 221, 259, 294); eosinopenia (d0, 139, 164, 221, 294); basopenia (d0, 221, 294); hypoglycemia (d164); ALT ↑ (d294).

Days after vaccination with rVSVΔG-NiV G or control (rVSVΔG-EBOV76-GP) vectors are in parentheses. All reported findings are in comparison to baseline (8 days prior to vaccination) values. Decreased appetite is defined as some food but not all food consumed from the previous day. Anorexia is defined as no food consumed from the previous day. Fever is defined as a temperature more than 2.5 °F over baseline, or at least 1.5 °F over baseline and ≥ 103.5 °F. Hypothermia is defined as a temperature ≤3.5°F below baseline. Lymphocytopenia, monocytopenia, erythrocytopenia, thrombocytopenia, neutropenia, eosinopenia, and basopenia are defined by a ≥35% drop in numbers of lymphocytes, monocytes, erythrocytes, platelets, neutrophils, eosinophils, or basophils, respectively. Lymphocytosis, monocytosis, neutrophilia, eosinophila, and basophilia are defined by a 100% or greater increase in numbers of lymphocytes, monocytes, neutrophils, eosinophils, and basophils, respectively. Hyperglycemia is defined as a 100% or greater increase in levels of glucose. Hypoglycemia is defined by a ≥25% decrease in levels of glucose. Anemia is defined as a concurrent ≥25% decrease in erythrocyte count, Hct, and Hgb. Hypoalbuminemia is defined by a ≥25% decrease in levels of albumin. Hypoproteinemia is defined by a ≥25% decrease in levels of total protein. Hypoamylasemia is defined by a ≥25% decrease in levels of serum amylase. Hypocalcemia is defined by a ≥25% decrease in levels of serum calcium. Increases in ALT, AST, ALP, CRE, CRP, Hct, and Hgb were graded on the following scale: ↑ = 1-5 fold, ↑↑ = >5-10 fold, ↑↑↑ = >10-20 fold, ↑↑↑↑ = >20-fold, ↓ = ≥50% decrease. (AGMs) African green monkeys, (BUN) blood urea nitrogen, (ALT) alanine aminotransferase, (AST) aspartate aminotransferase, (ALP) alkaline phosphatase, (CRE) Creatinine, (CRP) C-reactive protein, (Hct) hematocrit, (Hgb) hemoglobin.

Supplemental References

S1. Wickham H. *Ggplot2: Elegant Graphics for Data Analysis*. Springer-Verlag; 2018.

S2. Xu S, et al. Use *ggbreak* to effectively utilize plotting space to deal with large datasets and outliers. *Front Genet*. 2021;12:774846.

S3. Rvision - Colorblind-Friendly Color Maps for R. R package version 0.6.2. Version 0.6.2. Garnier S, et al.; 2021. https://sjmgarnier.github.io/viridis/.
